# Cryo-EM reveals a double oligomeric ring scaffold of the CHIKV nsP3 peptide in complex with the NTF2L domain of host G3BP1

**DOI:** 10.1128/mbio.03967-24

**Published:** 2025-04-11

**Authors:** Yuanzhi Liu, Jie Wang, Yinze Han, Xiaoyan Xia, Rui Zeng, Xinyu Fan, Bo Zhang, Kaituo Wang, Jian Lei

**Affiliations:** 1 National Clinical Research Center for Geriatrics, and State Key Laboratory of Biotherapy, West China Hospital, Sichuan University 660068 , Chengdu, Sichuan, China; 2 State Key Laboratory of Plant Diversity and Specialty Crops, Institute of Botany, Chinese Academy of Sciences 53033, Beijing, China; 3 Key Laboratory of Special Pathogens and Biosafety, Wuhan Institute of Virology, Center for Biosafety Mega-Science, Chinese Academy of Sciences 74614 , Wuhan, Hubei, China; Duke University School of Medicine, Durham, North Carolina, USA

**Keywords:** alphavirus, non-structural protein, stress granule, virus–host interaction, innate immune response

## Abstract

Chikungunya virus (CHIKV) poses a severe threat to global public health. The interaction between CHIKV nsP3 and host G3BP1 is critical for viral replication. However, the exact structural mechanism of the nsP3–G3BP1 interaction is scarce. Here, we report a cryo-electron microscopy structure of an octameric-heterotrimer formed by CHIKV nsP3 peptide (designated as CHIKV-43) in complex with the nuclear translocation factor 2-like (NTF2L) domain of G3BP1. The overall structure presents a double-layer ring scaffold. Two FGDF motifs and two alpha helices of CHIKV-43 are essential to bind NTF2L. Particularly, the secondary alpha helix plays key roles in forming the CHIKV-43–NTF2L high-order oligomer. We next analyzed the detailed interactions between CHIKV-43 and the NTF2L domain. The different binding patterns of NTF2L with its various partners were described as well. Subsequently, we demonstrated that the CHIKV-43 peptide is a crucial factor for nsP3 co-localization with G3BP1, reducing stress granule formation and interfering with interferon production. Overall, our findings present the structural and functional mechanisms on CHIKV nsP3 modulating host G3BP1 and provide a potential antiviral target based on the protein–protein interaction interface.

**IMPORTANCE:**

Chikungunya virus (CHIKV) is an arbovirus responsible for causing fever, headache, and severe joint pain in humans, with widespread outbreaks affecting millions worldwide. The CHIKV nsP3 is a key protein that interacts with multiple host proteins. In this study, we present the cryo-electron microscopy structure of a high-order oligomer formed by the CHIKV nsP3 peptide and the nuclear translocation factor 2-like (NTF2L) domain of host protein G3BP1, revealing a completely novel interaction model. The detailed interactions within this oligomer were illustrated. We also analyzed the binding patterns of the NTF2L domain of G3BP1 with its various partners, providing essential insights for the development of peptide-mimetic inhibitors targeting nsP3 and/or G3BP1. Furthermore, our data indicate that the nsP3–G3BP1 interaction reduces stress granule formation and antagonizes interferon production. Overall, this study provides new knowledge on CHIKV biology and suggests a potential target for developing antiviral therapeutics.

## INTRODUCTION

Chikungunya virus (CHIKV) is a positive-sense, single-stranded RNA virus transmitted by mosquito vectors (*Aedes aegypti* and *Aedes albopictus*). The outbreaks of CHIKV have occurred globally, including Africa, Asia, the Indian and Pacific Oceans, Europe, and America (https://www.cdc.gov/). The typical symptoms of people infected with CHIKV are fever and joint pain (1, 2). Other symptoms contain skin rash, muscle pain, headache, and joint swelling. 30% - 70% of CHIKV-infected patients appear in constant joint pain for months or even years (3). Furthermore, neonates and older individuals with preexisting conditions have reported death after infection with CHIKV (4–6). Currently, CHIKV infections are often misdiagnosed due to overlapping symptoms with Zika and dengue viruses (7, 8). The threat of CHIKV on public health is significantly underestimated. The first CHIKV vaccine (Ixchiq [9–11]) was just approved by the FDA; however, vaccination rates remain very low, and the outbreak of CHIKV is periodic, thus developing anti-CHIKV drugs is imperative in the clinic. Fundamental research on CHIKV is crucial for identifying potential antiviral targets.

CHIKV is a member of the Old World alphaviruses within the *Togaviridae* family (alphaviruses are classified into New World and Old World groups based on their disease characteristics and primary areas of endemicity). The genome of CHIKV is about 11.8 kb, consisting of a 5´ cap structure, two open reading frames (ORFs), and a 3´ polyadenylated tail. Two ORFs encode non-structural proteins (nsP1, nsP2, nsP3, and nsP4) and structural proteins (capsid, envelope protein [E1–E3], 6K, and transframe [TF]), respectively. The nsPs compose the viral replication complex to facilitate viral genome synthesis (12–14). The structural proteins act in nucleocapsid formation and virion assembly (15). CHIKV nsP3 plays multiple roles in viral replication and host modulation (16). It is divided into three domains: an N-terminal macrodomain (Macro), a central zinc-binding domain (ZBD, also designated as alphavirus unique domain), and a C-terminal hypervariable domain (HVD). Macro possesses ADP-ribosylhydrolase activity; it activates the protease activity of nsP2 (17, 18). ZBD is critical for viral RNA replication (19). Very recently, Kril et al. reported that CHIKV nsP3 polymerizes through its ZBD domain, forming tubular scaffolds that are crucial for infection (20). The C-terminal HVD has been reported to interact with various host proteins (21–27).

Ras GTPase-activating protein-binding protein (G3BP) is one of such host proteins to bind the HVD region (24–27). G3BP is a multi-domain protein, composed of the nuclear translocation factor 2-like (NTF2L) domain, the acidic domain, the proline-rich (P-rich) motif, the RNA recognition motif (RRM), and the C-terminal arginine-glycine-glycine-rich (RGG-rich) region. Three homologous proteins, G3BP1, G3BP2a, and G3BP2b, exist in humans. They have a similar protein domain architecture. G3BP1 plays a key role in the assembly and dynamics of host stress granules (SGs) (28). Notably, G3BP1 can inhibit RNA virus replication through stimulating SG formation (29, 30). Also, it recruits antiviral proteins to enhance innate immune response (31–34). Owing to the essential antiviral roles of G3BP1, different viruses employ various strategies to antagonize the function of G3BP1. For example, picornaviruses digested G3BP1 through their viral protease to inhibit the formation of SG (35, 36). Severe acute respiratory syndrome coronavirus 2 (SARS-CoV-2) nucleocapsid (N) protein directly binds to G3BP1 to counteract SG formation (37, 38).

Old World alphaviruses, including Semliki Forest virus (SFV) and CHIKV, have also evolved ways to prevent SG formation by viral nsP3 binding to G3BP1 (24, 39, 40). Disrupting the nsP3–G3BP1 interaction significantly attenuates the replication and proliferation of SFV and CHIKV (26, 40, 41). Panas et al. proved that nsP3 interacts with G3BP1 through its two FGDF (Phe–Gly–Asp–Phe) motifs localized in the HVD region (24). Crystal structures of single SFV FGDF motif (the N-terminal motif) and both FGDF motifs (named as SFV nsP3-25, containing a 25-residue peptide) in complex with the NTF2L domain of G3BP1/2 were reported (25, 26). The FGDF motif interacts with the typical hydrophobic pocket of the NTF2L region. Particularly, in the latter structure, Schulte et al. reported a possible poly-complex of G3BP1 dimers interconnected by SFV nsP3-25 during crystallization (26). In addition, a central helix (designated as alpha helix 1 [α1] in our study) between the two FGDF motifs is identified. According to the hypothetical model, these authors supposed that the corresponding α1 in CHIKV contributes significantly less to the binding of G3BP1 than that of nsP3 in SFV. However, the exact structural information on the CHIKV nsP3–G3BP1 complex has not yet been elucidated.

In this study, we presented the structure of the CHIKV nsP3 peptide (named as CHIKV-43) in complex with the NTF2L domain of G3BP1 for the first time. CHIKV-43 interacts with the NTF2L domain to form a high-order oligomer within a double-layer ring scaffold. The CHIKV-43 adopts two distinct conformations. The two FGDF motifs (motifs 1 and 2) and the alpha helix following each motif (α1 and α2) in CHIKV-43 play important roles in binding to G3BP1. Particularly, the α2 is an essential factor in stabilizing this oligomer, which is also illustrated for the first time. The detailed interactions between CHIKV-43 and NTF2L were described. We then analyzed the different binding patterns of the NTF2L domain with its various partners. We confirmed that the CHIKV-43 peptide is a key factor for co-localization with G3BP1. The nsP3–G3BP1 interaction disrupts the SG formation, leading to the reduction of interferon (IFN) production. Overall, our results provide new structural information on CHIKV nsP3 interacting with G3BP1 and suggest a potential antiviral target based on the structure interface between nsP3 and G3BP1.

## RESULTS

### A novel alpha helix of CHIKV nsP3 is a crucial factor for interacting with G3BP1 to form a high-order oligomer

**Fig 1:**
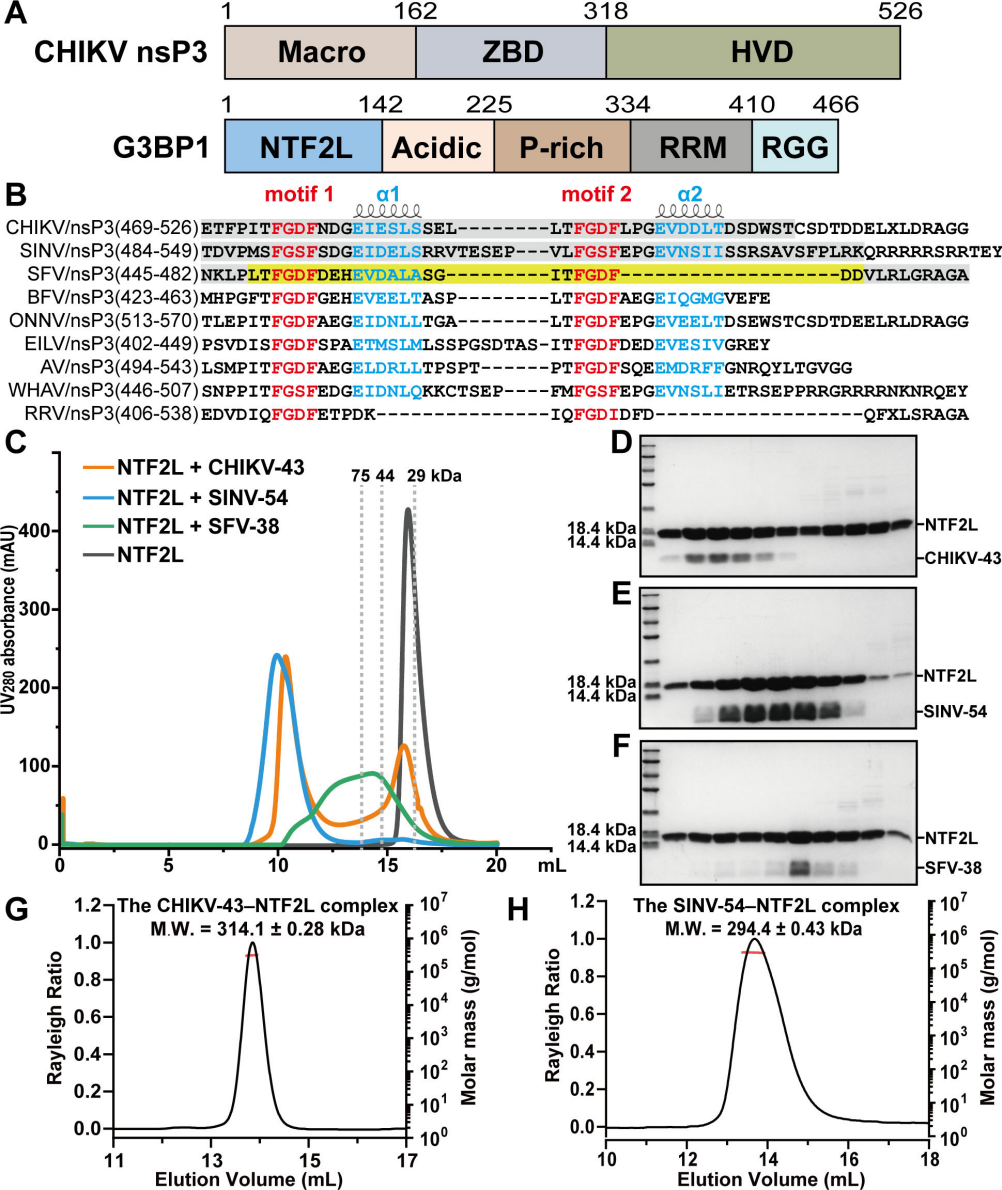
High-order oligomer of alphavirus nsP3 interacting with host G3BP1. (A) Domain architecture of CHIKV nsP3 (Asian strain JC2012, GenBank: AGJ84081.1) and host G3BP1 (NCBI Reference Sequence: NM_005754.3), with individual domains color coded. (B) Sequence alignment of the nsP3 HVD regions in Old World alphaviruses. The conserved G3BP-binding motifs (motifs 1 and 2) are annotated in red font. Two alpha helices (α1 and α2) of HVD are labeled in blue font. The sequences of CHIKV-43, SINV-54, and SFV-38 are boxed in gray. SFV nsP3-25 sequence (PDB code: 5FW5, [26]) is boxed in yellow. CHIKV, Chikungunya virus (AGJ84081.1); SINV, Sindbis virus (NP_062888.1); SFV, Semliki Forest virus (NP_463457.1); BFV, Barmah Forest virus (NP_818997.1); ONNV, O’nyong’nyong virus (NP_740705.1); EILV, Eilat virus (YP_006732326.1); AV, Aura virus (NP_819014.1); WHAV, Whataroa virus (YP_005351236.1); RRV, Ross River virus (NP_740680.1). X has been used for marking the residue that is generated from the leaky stop codon. (C) Size-exclusion chromatography (SEC) profiles of NTF2L alone and NTF2L incubated with CHIKV-43, SINV-54, and SFV-38, respectively. The peak positions of three standard proteins are shown as gray dashed lines. (D–F) SDS-PAGE analysis showing the CHIKV-43–NTF2L (D), SINV-54–NTF2L (E), and SFV-38–NTF2L (F) complexes. (G and H) SEC with multi-angle static light scattering analysis of the CHIKV-43–NTF2L (G) and SINV-54–NTF2L (H) complexes. M.W., molecular weight.

CHIKV nsP3 comprises the Macro, the ZBD, and the HVD, while host G3BP1 contains the NTF2L domain, the acidic domain, the P-rich motif, the RRM, and the C-terminal RGG-rich region (Fig. 1A). The NTF2L domain of G3BP1 has been verified to bind to various partners through the FGxF (Phe–Gly–x–Phe; x: Asp, Glu, or Ser) motif (24, 26, 42). By performing multiple sequence alignment of the HVD region of nsP3 in Old World alphaviruses, we found that almost all nsP3 proteins contain two canonical FGxF motifs, namely, motifs 1 and 2 (Fig. 1B). The crystal structure of the SFV nsP3-25 peptide (residues Leu449–Asp473, including two FGDF motifs) in complex with the NTF2L domain has been reported (26). Two FGDF motifs are important for binding to the NTF2L dimer. Unexpectedly, an alpha helix (residues _458_EVDALA_463_, referred to as α1), following motif 1, also plays an essential role in binding to NTF2L (26) (Fig. S1A). Of note, we identified another alpha helix (we designated it as α2, following motif 2) through secondary structure analysis (Fig. 1B; Fig. S1B through D). This α2 is present in CHIKV, Sindbis virus (SINV), Barmah Forest virus (BFV), etc., but is absent in SFV and Ross River virus (RRV). Additionally, the residues of the α2 (_500_EVDDLT_505_) in CHIKV are similar to those of α1 (_458_EVDALA_463_) in SFV (Fig. 1B). Given the importance of the SFV α1 in binding to the NTF2L domain, it is interesting to investigate whether the α2 in CHIKV plays any role in interacting with NTF2L, and whether there are various binding modes between nsP3 and NTF2L across different alphaviruses.

Thus, we first expressed and purified nsP3 peptides of CHIKV (residues Glu469–Thr511, named as CHIKV-43), SINV (Thr484–Lys537, named as SINV-54), and SFV (Asn445–Ala482, named as SFV-38) (Fig. 1B; Fig. S1E through G). The CHIKV-43 and SINV-54 peptides include two FGDF motifs and the α1 and α2, whereas the SFV-38 does not possess the α2 part (Fig. 1B). We next prepared the NTF2L domain (residues Met1–Phe138, Fig. S1H). Each peptide was mixed with NTF2L and subjected to size-exclusion chromatography (SEC) experiments (Fig. 1C). The results showed that the peak position of each mixture was obviously shifted left, compared to that of NTF2L alone. The SDS-PAGE results indicated the presence of the corresponding peptide and NTF2L in the elution fraction derived from the mixture peak (Fig. 1D through F). Notably, the mixture of CHIKV-43 (or SINV-54) and NTF2L formed a stable peak. Based on the SEC with multi-angle static light scattering (SEC-MALS) assays, the molecular weights (M.W.) of the CHIKV-43–NTF2L and SINV-54–NTF2L complexes are approximately 310 kDa (Fig. 1G) and 290 kDa, respectively (Fig. 1H). In contrast, the SFV-38–NTF2L mixture showed an irregular, wide-range peak (Fig. 1C). Additionally, the protein solution became turbid during the preparation of the SFV-38–NTF2L mixture. We suspected that these observations are likely due to the absence of the α2 in SFV-38.

**Fig 2:**
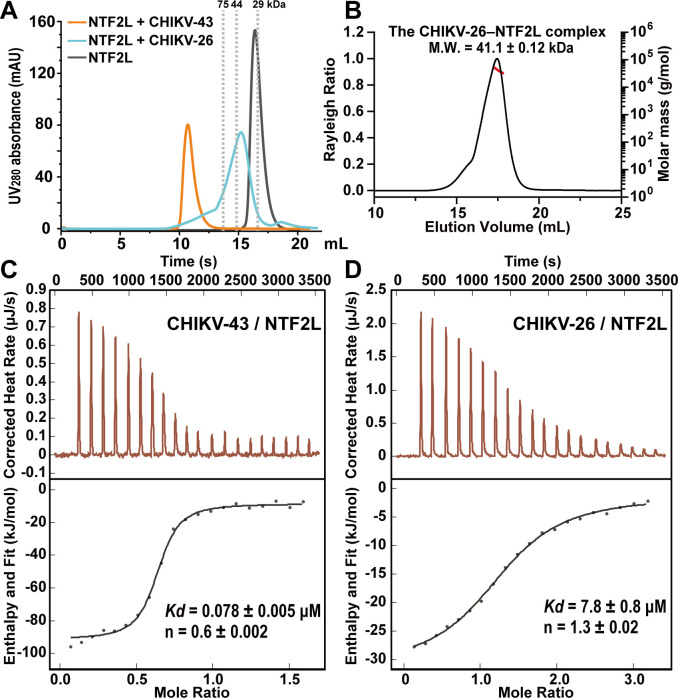
The secondary alpha helix region is crucial for the CHIKV-43–NTF2L high-order oligomer formation. (A) SEC profiles of NTF2L alone and NTF2L incubated with CHIKV-43 and CHIKV-26, respectively. The peak positions of three standard proteins are shown as gray dashed lines. (B) SEC-MALS analysis of the CHIKV-26–NTF2L complex. (C) Affinity between CHIKV-43 and NTF2L. (D) Affinity between CHIKV-26 and NTF2L. *Kd*, dissociation constant. n, molar ratio.

To verify our hypothesis, CHIKV-26 peptide (residues Ile473 to Pro498, without the α2 region) of CHIKV nsP3 was prepared and mixed with the NTF2L protein. Compared to the CHIKV-43–NTF2L complex, we found that the CHIKV-26 peptide and NTF2L cannot form the high-order oligomer (Fig. 2A). The M.W. of the CHIKV-26–NTF2L complex becomes approximately 40 kDa (Fig. 2B). Moreover, NTF2L and CHIKV-43 with point mutations in the α2 region cannot form the high-order oligomer either (see below). In addition, based on our isothermal titration calorimetry (ITC) assays, the binding affinity of CHIKV-26 with NTF2L is reduced approximately 100-fold, compared to that of CHIKV-43 with NTF2L (Fig. 2C and D). Meanwhile, we noticed that the binding molar ratio between CHIKV-43 and NTF2L is about 1:2, while the corresponding value is 1:1 between CHIKV-26 and NTF2L. These observations suggest that the α2 region does play a key role in the formation of the CHIKV-43–NTF2L oligomeric complex.

### Cryo-electron microscopy (cryo-EM) structure of CHIKV-43 in complex with the NTF2L domain of G3BP1

**Fig 3:**
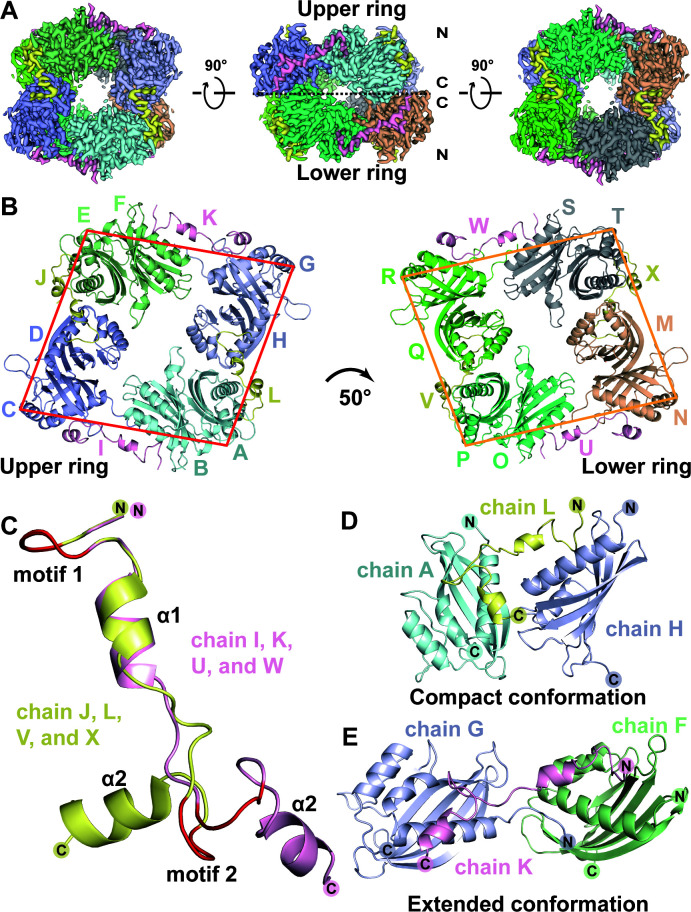
Overall structure of CHIKV-43 in complex with the NTF2L domain. (A) Cryo-EM density map of the CHIKV-43 peptide (light pink and pale yellow) and the NTF2L dimer (pale cyan, light blue, pale green, blue white, wheat, green cyan, chartreuse, and gray) complex. (B) Cartoon presentation of the CHIKV-43–NTF2L complex. (C) Structural overlay of two distinct conformations (chains J, L, V, and X vs I, K, U, and W) of the CHIKV-43 peptides. Cartoon views of chain J (in pale yellow) and I (in light pink). The FGDF motifs are colored in red. (D and E) Cartoon presentations of the compact (D) and extended (E) conformations of the CHIKV-43–NTF2L complex. All figures were generated using PyMOL (http://www.pymol.org).

The fresh proteins of CHIKV-43, SINV-54, and SFV-38 in complex with NTF2L were evaluated using the negative-staining electron microscopy method. The overall shape of the former two complexes resembles a ring, but no structured protein particles were found in the latter complex (Fig. S2A through C). The CHIKV-43–NTF2L complex structure was subsequently determined using the single-particle cryo-EM method to a resolution of approximately 2.7 Å (Fig. 3A; Fig. S2D and S3A). The structure of the NTF2L domain (PDB entry: 5FW5 [26]) was used as an initial model, and the CHIKV-43 peptides were then manually built. The final model consists of eight CHIKV-43 peptides and eight NTF2L homodimers (Fig. 3A). The theoretical M.W. of this complex is about 304 kDa, which is close to the result obtained from the SEC-MALS analysis (Fig. 1G). In the final model, the N-terminal residues Glu469–Phe471 and the C-terminal residues Trp509–Thr511 (or Ser510–Thr511) of the CHIKV-43 peptide cannot be built due to the absence of electron density (Fig. S3B and C). The complete NTF2Ls (residue 1–138) were built in four chains, C, G, N, and R, while other chains lack residues Met1–Glu4 owing to incomplete electron density map. Three alpha helices (αI–III) and five β-strands (βI–V), except for a loop region (Gly43–Asp53), of the NTF2L domain fit well into the map (Fig. S3D through N).

Totally, three continuous yet distinguishable conformations (referred to as Conformations I, II, and III) of the CHIKV-43–NTF2L complex were identified, accounting for 49.1%, 32.9%, and 18.0% of the total particles, respectively (Fig. S2D; Table S1). Conformation I represents an intermediate state between Conformations II and III (Fig. S4). These three conformations could interconvert to each other in solution (Fig. S4; Video S1).

Since the overall folds and interaction surfaces of the three conformations are nearly identical, we used Conformation I, which has the highest proportion, to illustrate the details. The overall structure of the CHIKV-43–NTF2L complex consists of a double-ring architecture (Fig. 3A). Each ring contains four NTF2L dimers and four CHIKV-43 peptides, presented in a parallelogram shape (Fig. 3B). The N-termini of all NTF2L chains faced outward, while the C-termini were oriented face-to-face (Fig. 3A). The two parallelograms displayed a rotation angle of approximately 50° (Fig. 3B). NTF2L forms the vertices of these two parallelograms; for simplicity, we designated these two quadrilaterals as “ACEG” (in the upper ring) and “NPRT” (in the lower ring) using the chain IDs of NTF2L (Fig. 3B).

The parallelogram “ACEG” was illustrated to reveal the binding mode between CHIKV-43 and NTF2L proteins. The CHIKV-43 peptide interacts with NTF2L at a molar ratio of 1:2, which is consistent with our ITC result (Fig. 2C). Four CHIKV-43 peptides can be divided into two groups based on two distinct conformations (Fig. 3C): chains L and J as well as chains K and I. Chain L (or J) interacting with two NTF2L protomers presents a compact conformation (Fig. 3D), while chain K (or I) binding to two NTF2Ls forms an extended conformation (Fig. 3E). The FGDF motifs 1 and 2 and the α1 and α2 of CHIKV-43 were involved in binding to the NTF2L molecules (Fig. 3D and E); the detailed interactions were described in the next section.

### Detailed analysis of the CHIKV-43 peptide interacting with the host NTF2L domain

**Fig 4:**
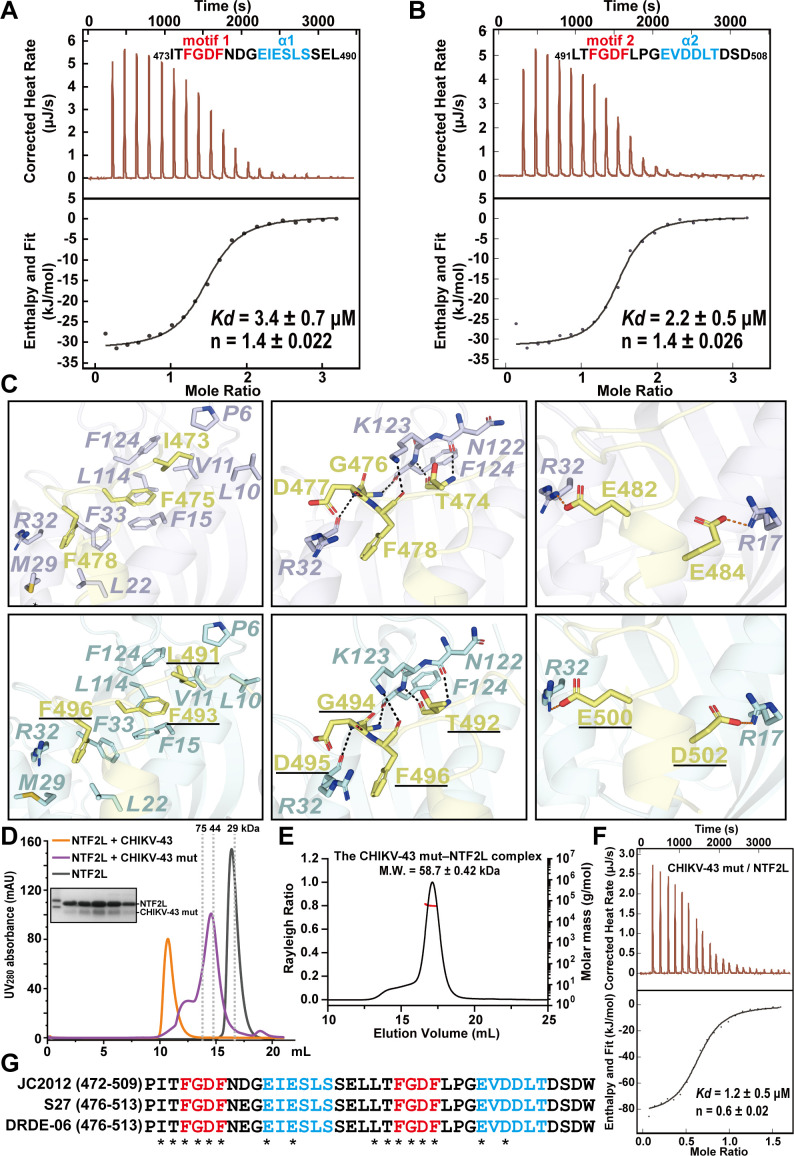
Structural analysis of the CHIKV-43–NTF2L complex. (A and B) Affinity between _473_ITFGDFNDGEIESLSSEL_490_ (A) or _491_LTFGDFLPGEVDDLTDSD_508_ (B) and NTF2L. *Kd*, dissociation constant. n, molar ratio. (C) Detailed interactions between CHIKV-43 and NTF2L in the compact conformation. H-bonds are indicated by black dashed lines. Ionic bonds are displayed by red dashed lines. (D) SEC profiles of NTF2L alone and NTF2L incubated with CHIKV-43 and CHIKV-43 mut, respectively. The peak positions of three standard proteins are shown as gray dashed lines. (E) SEC-MALS analysis of the CHIKV-43 mut–NTF2L complex. (F) Affinity between CHIKV-43 mut and NTF2L. (G) Sequence alignment of the CHIKV-43 peptides among Asian strain JC2012 (GenBank: AGJ84081.1), African strain S27 (AAN05101.1), and Indian strain DRDE-06 (ABP88821.1). Residues involved in binding to NTF2L are absolutely conserved. They are marked with asterisks. Panel C was generated using PyMOL (http://www.pymol.org).

The interface between CHIKV-43 and the NTF2L domain is mediated by hydrogen (H) bonds, hydrophobic interactions, and ionic interactions. The N-terminal region (_473_ITFGDFNDGEIESLSSEL_490_, containing the motif 1 and α1) and C-terminal region (_491_LTFGDFLPGEVDDLTDSD_508_, including the motif 2 and α2) of CHIKV-43 displayed almost the same binding mode to NTF2L. Their binding affinities with NTF2L are 3.4 ± 0.7 and 2.2 ± 0.5 µM, respectively (Fig. 4A and B). The detailed interactions between CHIKV-43 and NTF2L were illustrated based on N- and C-terminal regions of the CHIKV-43 peptide.

Taking the compact conformation (Fig. 3D) as an example, the side chain of Ile473 (in the N-terminal region) or Leu491 (in the C-terminal region, residue is underlined and as follows) inserts into the hydrophobic cavity formed by the side chains of *Pro6*, *Leu10*, *Val11*, and *Phe124* in NTF2L (residues of NTF2L are in italics and as follows, Fig. 4C, left panels, Table S2). The main-chain nitrogen and oxygen atoms of Thr474 (or Thr492) make H-bonds with *Asn122* and *Phe124*, respectively (Fig. 4C, middle panels, Table S2). In the _475_FGDF_478_ and _493_FGDF_496_ motifs, the aromatic ring of Phe475 (or Phe493) makes hydrophobic contacts with *Val11*, *Phe15*, *Phe33*, *Leu114*, and *Phe124*, and the aromatic ring of Phe478 (or Phe496) is stabilized via hydrophobic interactions with the side chains of *Leu22*, *Met29*, *Arg32*, and *Phe33* (Fig. 4C, left panels, Table S2). The main-chain nitrogen atom of Gly476 (or Gly494) forms an H-bond with the backbone oxygen of *Phe124* (Fig. 4C, middle panels, Table S2). Meanwhile, the main-chain nitrogen atom of Asp477 (or Asp495) and the backbone oxygen of *Arg32*, as well as the main-chain oxygen atom of Phe478 (or Phe496) and the side chain of *Lys123*, make up additional H-bonds (Fig. 4C, middle panels, Table S2). Following each FGDF motif, two alpha helices (α1 and α2) are also involved in binding to NTF2L. Glu482 and Glu484 of α1 interact with *Arg32 and Arg17* in NTF2L through ionic interactions, respectively (Fig. 4C, right-upper panel, Table S2). The interaction model involving two motifs and α1 between CHIKV-43 and NTF2L is consistent with the observations as reported in SFV (26). However, we noticed that Glu500 and Asp502 of α2 make another two ionic bonds with *Arg32 and Arg17* of NTF2L (Fig. 4C, right-lower panel, Table S2). In particular, Glu500 is completely conserved in the other Old World alphaviruses that contain the α2 (Fig. 1B). To clarify the role of these two salt bridges, both residues Glu500 and Asp502 were mutated to Ala (designated as CHIKV-43 mut, Fig. S1I). Of note, CHIKV-43 mut and NTF2L mainly form a complex with the M.W. of about 60 kDa (Fig. 4D and E). The ITC assay showed that the binding affinity between CHIKV-43 mut and NTF2L is ~15-fold weaker than that of CHIKV-43 and NTF2L (Fig. 2C and 4F). These results indicate that Glu500 and Asp502 are crucial for the CHIKV-43 binding to NTF2L and, once again, suggest that α2 plays a key role in the formation of the CHIKV-43–NTF2L high-order oligomer.

In the extended conformation structure (Fig. 3E), the interface between CHIKV-43 and NTF2L is exactly the same as that in the compact conformation structure (Fig. S5). Further sequence alignment of CHIKV-43 in the Asian strain JC2012, African strain S27, and Indian strain DRDE-06 revealed that the residues of CHIKV-43 involved in binding to NTF2L are absolutely conserved (Fig. 4G), indicating that the nsP3–NTF2L interaction mode is preserved across various CHIKV strains.

### Different interaction interfaces among the NTF2L domains

**Fig 5:**
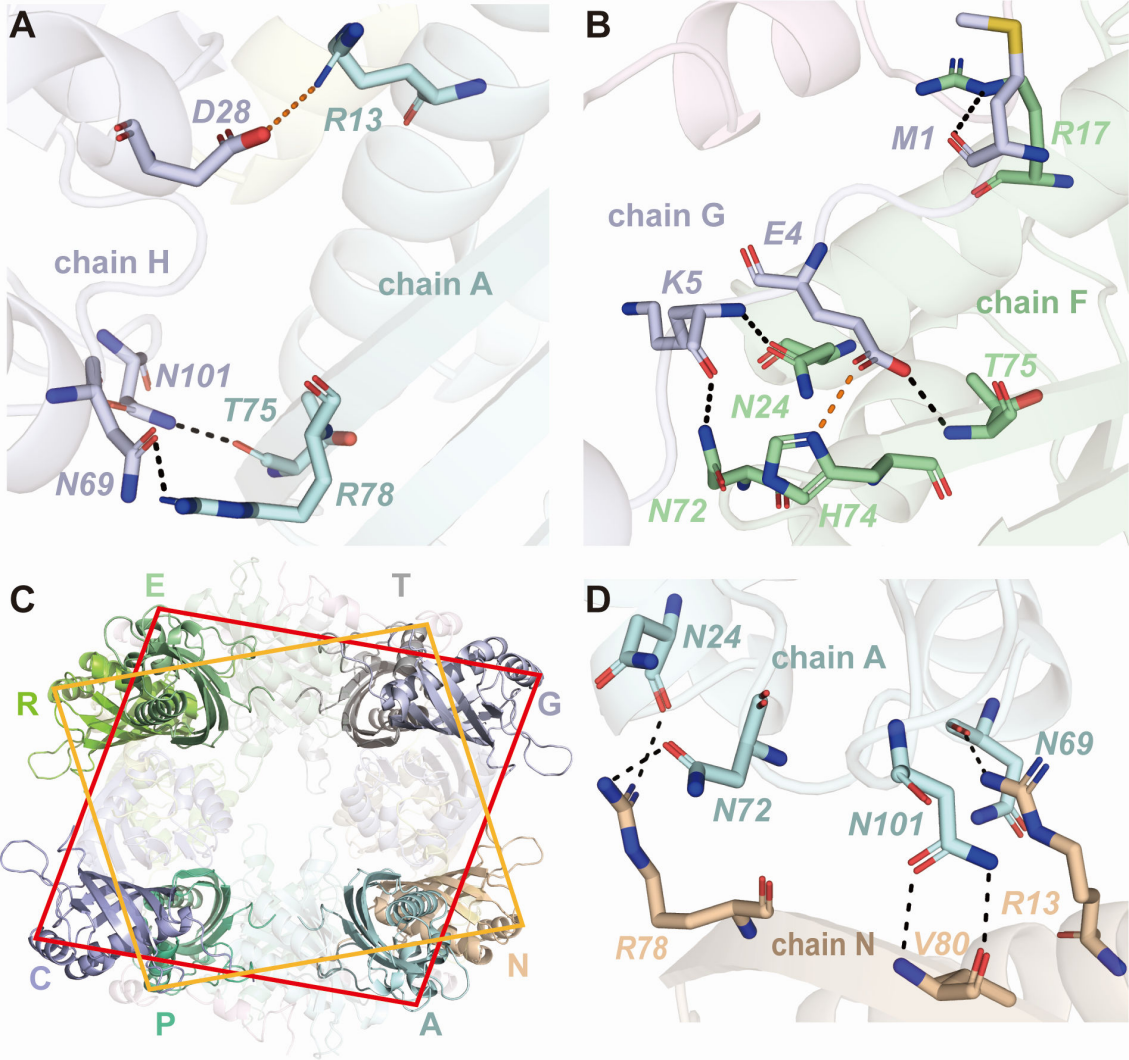
Interface analysis between the NTF2L domains. (A and B) Detailed interactions between adjacent NTF2L domains in the compact (A) and extended (B) conformations. H-bonds are indicated by black dashed lines. Ionic bonds are displayed by red dashed lines. (C) Double-layer ring scaffold stabilized by interfaces between chains A and N, C and P, E and R, as well as G and T. (D) Detailed interactions between chains A and N. H-bonds are indicated by black dashed lines. Panels were generated using PyMOL (http://www.pymol.org).

Based on the CHIKV-43–NTF2L complex structure, adjacent NTF2L dimers (in each ring) present two distinct interfaces. In the compact conformation (Fig. 3D), *Thr75* and *Arg78* in chain A make H-bonds with *Asn101* and *Asn69* in chain H (Fig. 5A; Table S3), respectively. *Arg13* in chain A makes ionic interaction with *Asp28* in chain H. In the extended conformation (Fig. 3E), the N-terminal residues (_1_MVMEK_5_) of one NTF2L domain (chain G) insert into the other NTF2L (chain F). *Met1* and *Glu4* in chain G form H-bonds with *Arg17* and *Thr75* in chain F, respectively. *Glu4* also makes an ionic bond with *His74*. Additionally, *Lys5* in chain G forms two H-bonds with *Asn24* and *Asn72* in chain F (Fig. 5B; Table S3).

Meanwhile, the NTF2Ls in the upper and lower rings also interact with each other. These interfaces are located at the vertices of the two parallelograms (AN, CP, ER, and GT) (Fig. 5C). Taking chains A (in the upper ring) and N (in the lower ring) as examples, five H-bonds are formed between these two chains (Fig. 5D; Table S3). They are *Asn24-Arg78*, *Asn69-Arg13*, *Asn72-Arg78*, and *Asn101-Val80* (two H-bonds). The same interactions exist in the other three interface regions, i.e., CP, ER, and GT; thus, a total of 20 H-bonds maintain the two-layer ring structure.

### Different binding modes between the NTF2L domain and its various partners

**Fig 6:**
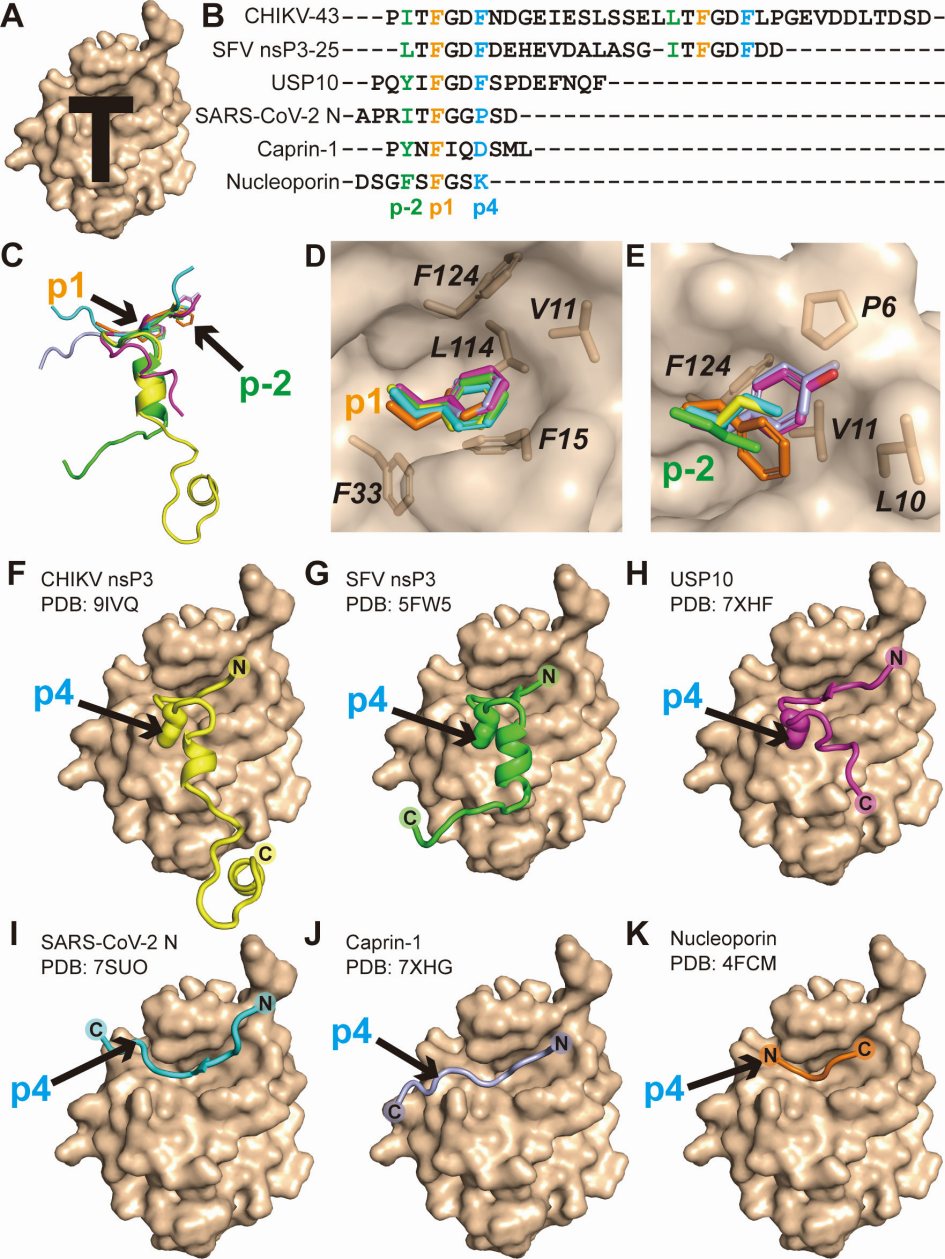
Structural comparison of NTF2L interacting with its various partners. (A) Surface representation of the NTF2L domain. “T-shaped” surface groove is used to bind partners. The surface of the NTF2L domain is colored in wheat. (B) Sequence alignment of the NTF2L-binding regions from its various partners: CHIKV nsP3, SFV nsP3, USP10, SARS-CoV-2 N protein, caprin-1, and nucleoporin. The p-2, p1, and p4 positions are colored in green, orange, and blue, respectively. (C) Superimposition of the NTF2L-binding partners, revealing conserved p-2 and p1 positions. Peptides of CHIKV nsP3, SFV nsP3, USP10, SARS-CoV-2 N protein, caprin-1, and nucleoporin are colored in yellow, green, magenta, cyan, light blue, and orange, respectively. (D) Phenylalanine at p1 position inserting into the hydrophobic cavity of NTF2L. (E) The side chains of hydrophobic residues at p-2 position inserting into the hydrophobic pocket of NTF2L. (F–K) “T-shaped” or “▬-shaped” conformation of the NTF2L-binding partners determined by residue at the p4 site. CHIKV nsP3 (F), SFV nsP3 (G), and USP10 (H) with phenylalanine at the p4 position present a “T-shaped” conformation. SARS-CoV-2 N (I), caprin-1 (J), and nucleoporin (K) with other residues at the p4 site exhibit a “▬-shaped” conformation. Panels A and C–K were generated using PyMOL (http://www.pymol.org).

Currently, five crystal structures of the NTF2L domain in complex with its partners, i.e., SFV nsP3 (26), ubiquitin-specific peptidase 10 (USP10) (42), SARS-CoV-2 N protein (38), cytoplasmic activation/proliferation-associated protein 1 (caprin-1) (42), and nucleoporin (43), have been reported. Typically, NTF2L presents a surface groove shaped like a letter “T,” formed by helices I and II and the loop connecting βIV–βV of the NTF2L domain (Fig. 6A). This groove is involved in binding to the FGxF motif. To investigate the binding patterns between NTF2L and its various partners, we performed a structure-based multiple-sequence alignment among different NTF2L-binding peptides (Fig. 6B). The results showed that the first phenylalanine (designated as p1 site, following the nomenclature from the literature [38]) of the FGxF motif is absolutely conserved. The p1-site Phe inserts into a hydrophobic cavity formed by the side chains of *Val11*, *Phe15*, *Phe33*, *Leu114*, and *Phe124* in NTF2L (Fig. 6C and D). The residues at position p-2 are completely hydrophobic; they bind to another hydrophobic pocket composed of *Pro6*, *Leu10*, *Val11*, and *Phe124* in NTF2L (Fig. 6C and E). However, the second phenylalanine (p4 site) of the FGxF motif is not strictly conserved. Based on our CHIKV-43–NTF2L structure, this Phe residue is located in the hydrophobic pocket formed by the side chains of *Leu22*, *Met29*, *Arg32*, and *Phe33* (Fig. 4C and 6F). Interestingly, the NTF2L-binding peptides of CHIKV nsP3, SFV nsP3, and USP10, which contain a phenylalanine at position p4, display a “T-shaped” fold (Fig. 6B and F through H). In contrast, the binding peptides of SARS-CoV-2 N, caprin-1, and nucleoporin, which have other residues at the p4 site, present a “▬-shaped” scaffold (Fig. 6B and I through K). The p4-site residue of the NTF2L-binding peptides appears to determine whether they adopt a “T-shaped” or “▬-shaped” conformation.

Collectively, these findings suggest that the residues at the p1 and p-2 sites of the NTF2L-binding partners interact with the conserved binding pocket of NTF2L, whereas the residue at the p4 position likely determines the binding pattern with NTF2L.

### CHIKV-43 is a key factor in modulating SG formation and interferon production

**Fig 7:**
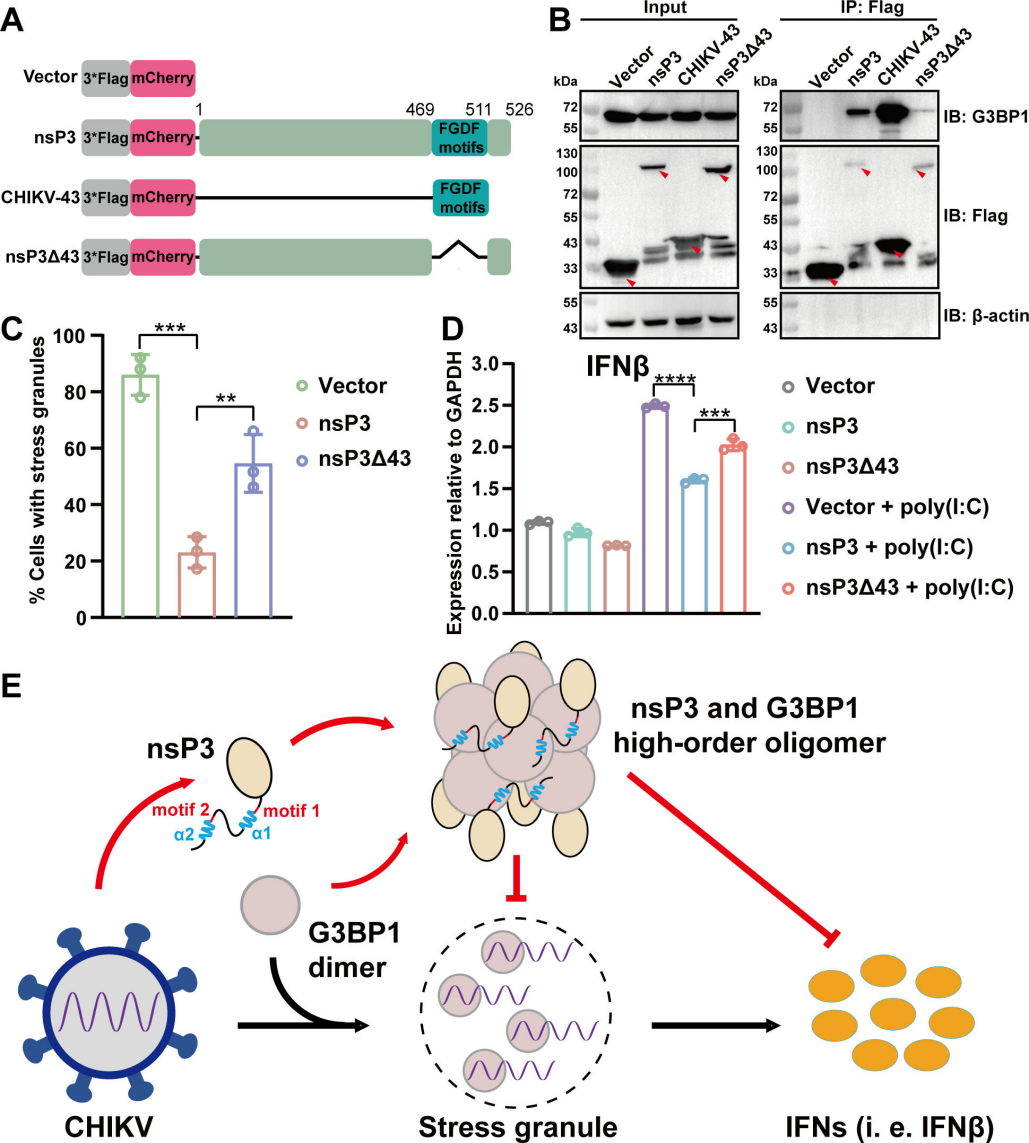
CHIKV-43 is an essential factor to interact with G3BP1 and regulate SG formation and IFN production. (A) Schematic diagram of CHIKV nsP3 and its truncations. (B) Co-IP analysis of the interaction between nsP3, its truncations, and endogenous G3BP1 in HEK293T cells. Red arrows indicate targeted proteins. (C) Quantitative analysis of SG percentage in HEK293T cells from Fig. S6B. The number of cells with SGs was analyzed for at least 50 cells per condition. (D) The impact of CHIKV nsP3 or nsP3Δ43 on IFNβ production. HEK293T cells were transfected with plasmids of control vector, nsP3, and nsP3Δ43, then cells were further stimulated with poly(I:C). Subsequently, cells were collected for real-time quantitative PCR analysis to determine the relative expression levels of IFNβ normalized by GAPDH. (C and D) All experiments were replicated three times. Error bars represent mean ± standard deviation, **P < 0.01, ***P < 0.001, and ****P < 0.0001 (Student’s *t*-test, *n* = 3). (E) The model of CHIKV nsP3 hijacking host G3BP1 to form a high-order oligomer and antagonize SG formation and interferon production. Two motifs (1 and 2) and the helices (α1 and α2) of nsP3 are marked.

Previous studies have shown that the nsP3 protein of alphaviruses inhibits SG formation by interacting with G3BP1 through the FGDF motif (24, 40). To further confirm the role of CHIKV-43 in this process, we performed co-immunoprecipitation (Co-IP) assays of nsP3, CHIKV-43 peptide, and nsP3Δ43 (the deletion of the CHIKV-43 peptide in nsP3) with endogenous G3BP1 in HEK293T cells, respectively (Fig. 7A). The results showed that nsP3 and CHIKV-43 obviously interact with G3BP1, while nsP3Δ43 displayed very weak interaction with G3BP1 (Fig. 7B). Consistent with these findings, nsP3Δ43 displayed weak co-localization with G3BP1 in immunofluorescence (IF) experiments (Fig. S6A), compared to the intact nsP3. In IF assays, nsP3 foci or G3BP1/nsP3 foci were observed, as reported in previous studies (39, 44). We then performed arsenite-induced SG formation assays in HEK293T cells transfected with control vector, nsP3, or nsP3Δ43 plasmids. Based on previous studies (27, 40), we used T cell-restricted intracellular antigen-1 (TIA-1) as an SG marker to distinguish SGs from G3BP1/nsP3 foci. The results displayed that SG formation was effectively reduced in the CHIKV nsP3 group, compared to the vector and nsP3Δ43 groups (Fig. 7C; Fig. S6B). SG assembly can stimulate the host innate immune response, primarily through upregulating the production of type I IFNs, such as IFNβ (31, 33, 37). We proved that CHIKV-43 is essential for modulating SG formation, leading us to hypothesize that this peptide may affect interferon production. To test this hypothesis, we stimulated HEK293T cells with poly(I:C), transfected with control vector, nsP3, or nsP3Δ43 plasmids. Compared to the vector and nsP3Δ43 groups, we found that the transcription level of IFNβ was significantly reduced in the nsP3 group (Fig. 7D).

Overall, the above results demonstrate that the CHIKV-43 peptide of nsP3 is essential for binding to G3BP1 and plays an important role in nsP3-mediated reduction of SG formation and interferon production.

## DISCUSSION

In our study, we revealed that the CHIKV-43 peptide of nsP3 binds to the NTF2L domain of G3BP1, forming a high-order oligomer with a completely novel binding scaffold. Two FGxF motifs (motifs 1 and 2) and two alpha helices (α1 and 2) are involved in binding to NTF2L. Particularly, the α2 is a key factor in stabilizing this oligomer. The nsP3–G3BP1 interaction not only disrupts SG formation but also inhibits host interferon production in cell assays. Based on our results, we proposed a model to illustrate how CHIKV nsP3 hijacks G3BP1 to form a high-order oligomer and modulate SG formation and the host innate immunity (Fig. 7E).

We observed three continuous yet distinguishable conformations of the CHIKV-43–NTF2L complex in solution. To investigate the cause of the slight conformational changes, we compared the NTF2Ls and CHIKV-43 peptides across three conformations (Fig. S7). The structures of NTF2Ls remain consistent in all conformations (e.g., chains A and B). However, the flexible loops between α1 and motif 2 in the CHIKV-43 peptides exhibit conformational variability. This suggests that the CHIKV-43 peptide may drive the observed differences. Whether these conformational changes have any biological significance requires further investigation.

According to our multiple-sequence alignment analysis, most nsP3 proteins in the Old World alphaviruses, such as SINV, BFV, and O’nyong’nyong virus, etc., contain two FGxF motifs and the α1 and 2 helices. Like CHIKV, these viruses could utilize the similar mechanism of nsP3 interacting with G3BP1. However, some alphaviruses, for example, SFV and RRV, do not include the α2; even α1 is absent in RRV, suggesting their nsP3 adopts a different mechanism on the nsP3–G3BP1 interaction. It is interesting to investigate why these alphaviruses exhibit the different nsP3–G3BP1 binding fashions in the viral life cycle. In our study, the N-terminus (motif 1 and α1) and C-terminus (motif 2 and α2) of CHIKV-43 exhibited similar binding affinities to NTF2L. In contrast, previous research on the SFV nsP3 peptide reported a significantly stronger binding of motif 1 to NTF2L compared to motif 2 (26). We hypothesize that the α1, adjacent to motif 1, may stabilize its conformation and enhance binding to NTF2L. In comparison, the absence of a corresponding α2 for motif 2 in SFV may hinder its ability to adopt a favorable conformation for binding. In addition, the CHIKV-26 and NTF2L form a complex with an approximate M.W. of 40 kDa and a 1:1 molar ratio (Fig. 2B and D). We speculate that this complex consists of two CHIKV-26 peptides and one NTF2L dimer (the theoretical M.W. is 38 kDa), which is consistent with our crystal structure of the CHIKV-26–NTF2L complex (PDB code: 9J5S). While the CHIKV-43 mut–NTF2L complex has an approximate M.W. of 60 kDa with a 1:2 molar ratio (Fig. 4E and F), it suggests that this complex comprises two CHIKV-43 mut peptides and two NTF2L dimers (the theoretical M.W. of ~76 kDa). A similar discrepancy between the SEC-MALS and theoretical M.W. values was also observed for the SFV nsP3-25–NTF2L complex (24).

A recent study reported that CHIKV nsP3 forms helical scaffolds (HSs) via its ZBD domain (20). In CHIKV-infected cells or cells stably expressing nsP3, these scaffolds organize into higher-order tubular structures (Fig. S8A). However, the mechanism driving this arrangement remains unclear. Transmission electron microscopy studies (20) show that the distance between two nsP3 HSs is approximately 12 nm–20 nm, while the width of our CHIKV-43–NTF2L cryo-EM structure is about 8 nm (Fig. S8A and B). This suggests sufficient space to accommodate the entire HVD region and G3BP1 between the HSs. Based on these findings, we hypothesize that our double-layered structure may link nsP3 HSs, facilitating the formation of higher-order tubular structures (Fig. S8C and D). Supporting this model, confocal and electron microscopy studies have demonstrated that CHIKV-nsP3 tubes incorporate host G3BPs (20). Nevertheless, further experimental validation is needed to confirm this hypothesis.

CHIKV nsP3 is a multiple-domain protein with the Macro, the ZBD, and HVD. We verified that CHIKV-43 of the HVD is a key region binding to G3BP1. However, the construct nsP3Δ43 still showed a weak interaction with G3BP1 (Fig. 7B), suggesting that other region(s) of nsP3 may be involved in binding to G3BP1. In our cell assays, we also noticed that nsP3Δ43 exhibits slight reduction in SG formation (Fig. 7C; Fig. S6B). A previous study reported that the macrodomain of CHIKV nsP3 can disrupt SG formation (45), which could partially explain our observations. It is worth investigating whether different domains of nsP3 play a synergistic role in modulating SG formation in the future.

G3BP1-driven SG formation not only affects viral replication (35–39, 46) but also plays roles in oncogenesis (47), cancer chemoresistance (48), and neurodegeneration occurrence (49). Thus, targeting G3BP1 to develop inhibitors is an essential therapeutic strategy. For example, two compounds (imatinib and decitabine) targeting G3BP1 have been utilized to inhibit SARS-CoV-2 propagation (50). Peptide analog GAP161, which targets G3BP1, has been proven to suppress colon carcinoma development (51). Epigallocatechin gallate inhibits lung cancer cell growth through targeting G3BP1 (52). By comparing the binding patterns of the NTF2L domain of G3BP1 with its different partners, we identified an essential “T-shaped” pocket of NTF2L and illustrated the key residue to determine the adopted binding mode of the NTF2L-binding peptides. These results provide useful structural information for the development of peptide-like inhibitors against G3BP1.

In conclusion, we reported a cryo-EM structure of a high-order oligomer formed by CHIKV nsP3 peptide (CHIKV-43) in complex with the NTF2L domain of G3BP1 for the first time. The α2 of CHIKV-43 plays a key role in stabilizing this oligomer. The detailed interaction interfaces between CHIKV-43 and NTF2L were described. Additionally, we illustrated the different binding patterns between NTF2L and its partners. Moreover, we demonstrated the important role of CHIKV-43 in binding to G3BP1, regulating SG formation, and antagonizing host interferon production. Collectively, our findings provide new structural insights into the interaction between CHIKV nsP3 and host G3BP1, as well as a potential antiviral target based on their interaction interface.

## MATERIALS AND METHODS

### Plasmid constructs

DNA fragment encoding human G3BP1-NTF2L domain (residues 1–138, NCBI Reference Sequence: NM_005754.3) was PCR-amplified from a HEK293T cDNA library. The cDNA library was created by Hifair III 1st Strand cDNA Synthesis SuperMix (YEASEN, 11184ES03). DNA fragment encoding G3BP1-NTF2L domain was inserted into the modified pET28a(+) plasmid containing an N-terminal hexahistidine (6xHis) tag and a tobacco etch virus (TEV) protease cleavage site. CHIKV-43 fragment (residues 469–511, GenBank: AGJ84081.1) adding C-terminal 6xHis tag was amplified by PCR using pCold TF-nsP3 plasmid (GENERAL BIOL., Anhui, China) and then cloned into the pGEX-6P-1 plasmid. The 6xHis tag was used to further purify the CHIKV-43 peptide. pGEX-6P-1-SINV (residues 484–537)−6xHis (SINV-54, NCBI Reference Sequence: NP_062888.1) and pGEX-6P-1-SFV (residues 445–482)−6xHis (SFV-38, NCBI Reference Sequence: NP_463457.1) were purchased from GENERAL BIOL., Anhui, China. The pGEX-6P-1-CHIKV-43 mut-6xHis was obtained by introducing mutations using the corresponding primers.

The mCherry-nsP3 fragment was obtained by overlapping extension PCR methodology. Briefly, overlapping fragments encoding mCherry and CHIKV nsP3 (residues 1–526, GenBank: AGJ84081.1) were PCR-amplified from pLVX-IRES-mCherry and pCold TF-nsP3 plasmids (GENERAL BIOL., Anhui, China). Two fragments were mixed in an equimolar ratio and used as a template to generate the mCherry-nsP3 fragment. This fragment was then cloned into the pCMV-3xFlag plasmid using the Hieff Clone Universal II One Step Cloning Kit (YEASEN, 10923ES20). Similarly, pCMV-3xFlag-mCherry-CHIKV-43 and pCMV-3xFlag-mCherry-nsP3Δ43 were constructed as described above, while pCMV-3xFlag-mCherry was used as the control vector.

All constructs were confirmed by sequencing (Tsingke Biotech Co., Ltd., Chengdu, China). The sequences of the primers used in this study are listed in Table S4.

### Protein expression and purification

The pET28a(+)-NTF2L plasmid was transformed into *Escherichia coli* BL21 (DE3) (Novagen). Then, the bacteria were cultured at 37°C in Luria-Broth medium supplemented with antibiotic and induced for 18 h with 0.5 mM isopropyl-β-D-thiogalactoside at 18°C. The expression culture was subsequently harvested by centrifugation, and the bacterial pellet was re-suspended in buffer A (20 mM Tris-HCl, 10 mM imidazole, 500 mM NaCl, 10% glycerol, pH 7.5) and lysed by sonication on ice. Debris was removed by centrifugation, and the supernatant was purified by the Ni-NTA QZT 6FF (Senhui Microsphere Technology Co., Ltd., Suzhou, China) with buffer A and buffer B (20 mM Tris-HCl, 500 mM imidazole, 500 mM NaCl, 10% glycerol, pH 7.5). Then, 6xHis-tagged NTF2L was digested overnight with TEV protease to remove the 6xHis tag (left with three extra residues Gly–Ala–Ser at the N-terminus after this processing step) and dialyzed against buffer C (20 mM Tris-HCl, 150 mM NaCl, 10% glycerol, pH 7.5) overnight at 4°C. The next day, the target protein was applied to Ni-NTA QZT 6FF again to remove the uncleaved 6xHis tag protein. NTF2L without the 6xHis tag was further purified by SEC (Superdex 75 Increase 10/300 GL, GE Healthcare) in buffer C. The quality of purified NTF2L was checked by SDS-PAGE.

The CHIKV-43, SINV-54, SFV-38, and CHIKV-43 mut were expressed using the same strategy as that of NTF2L (see above). Then, they were purified by GST QZT 4FF (Senhui Microsphere Technology Co., Ltd., Suzhou, China) with GST buffer (20 mM Tris-HCl, 500 mM NaCl, 10% glycerol, pH 7.5). Proteins were eluted with 10 mM glutathione (LABLEAD, GGGTH01) in GST buffer. The eluted proteins were incubated with PreScission protease at 4°C overnight to remove the GST tag (left with five residues Gly–Pro–Leu–Gly–Ser at the N-terminus). Cleaved peptides were further purified by the Ni-NTA QZT 6FF. These peptides were then purified by SEC (Superdex 75 Increase 10/300 GL, GE Healthcare) in buffer C. The quality of CHIKV-43, SINV-54, SFV-38, and CHIKV-43 mut was checked by SDS-PAGE.

Purified NTF2L protein was mixed with CHIKV-43, SINV-54, SFV-38, and CHIKV-43 mut, respectively. Each mixture was incubated at 4°C overnight and purified by SEC (Superdex 200 Increase 10/300 GL, GE Healthcare). The CHIKV-43–NTF2L complex was further purified by SEC (Superose 6 Increase 10/300 GL, GE Healthcare) for the cryo-EM experiment. Similarly, the NTF2L protein was mixed with CHIKV-26 peptide (synthesized by GL Biochem Ltd., Shanghai, China), then the mixture was purified by SEC (Superdex 200 Increase 10/300 GL, GE Healthcare).

### SEC-MALS

CHIKV-43–NTF2L, SINV-54–NTF2L, CHIKV-26–NTF2L, and CHIKV-43 mut–NTF2L complexes were concentrated to ~2 mg/mL in phosphate-buffered saline (PBS) (2.0 mM KH_2_PO_4_, 137 mM NaCl, 10.0 mM Na_2_HPO_4_, 2.7 mM KCl, pH 7.4). Each complex was injected into column Superdex 200 Increase 10/300 GL (GE Healthcare) with a flow rate of 0.5 mL/min. Protein in the eluent was detected via a Wyatt Dawn Heleos II Multi-Angle Light Scattering detector (Wyatt Technologies). The data were recorded and analyzed with Wyatt Astra software (version 8.1.2.1).

### Negative-staining electron microscopy

The CHIKV-43–NTF2L and SINV-54–NTF2L complexes were diluted to ~0.05 mg/mL in HEPES buffer (20 mM HEPES, 150 mM NaCl, pH 7.5), respectively. The mixture of SFV-38 and NTF2L was diluted to ~0.1 mg/mL in HEPES buffer.

Each sample (3 µL) was applied on the glow-discharged 300-mesh Cu grids (Quantifoil). The grid was washed three times with HEPES buffer and was then stained with uranyl acetate (3%, vol/vol). Excessive uranyl acetate was removed, and the grid was finally air-dried. Next, the grid was loaded into a JEM-1400 operated at 120 kV, condenser lens aperture 150 µm, spot size 1. Negative-staining images were captured using RADIUS software on a Morada G3 direct electron camera.

### Cryo-EM sample preparation

All cryo-EM samples were prepared using Vitrobot Mark VI operated at 100% humidity and 4°C. The grids (ANTcryo R 1.2/1.3 Cu 300 mesh) were glow discharged at 10 mA for 60 s (EM ACE200, Leica Microsystems). A 3-µL sample was applied to each grid and then incubated for 5 s and blotted for 3 s with blotting force 2. Prepared grids are stored in LN2 until data collection.

### Single-particle cryo-EM data collection, processing, model building, and refinement

The cryo-EM data sets of CHIKV-43–NTF2L were collected on Titan Krios electron microscopes (FEI) operated at 300 kV with a Falcon 4i detector in eer format. The pixel size was 0.725 Å and the total dose was 40e^-^ per Å^2^. An energy filter at 10 eV was applied during data collection. All data were processed using cryoSPARC v.3.4 (53) following the procedures outlined in Fig. S2D and Table S1. The data were initially processed using patch motion correction and patch contrast transfer function (CTF) determination. Blob particles with a diameter of 100 Å–140 Å were picked without templates and extracted to a small box size with bin2 for initial particle cleaning and 2D classification. The full-sized particles with box size of 320 pixels were re-extracted and processed following standard cryoSPARC workflow including 2D classification, *ab initio* model reconstitution, multiple rounds of heterogeneous refinement, and non-uniform refinement. The crystal structure of SFV nsP3-25 peptide with NTF2L dimer (PDB entry: 5FW5 [26]) was used as an initial model for model building. The final CHIKV-43–NTF2L complex structure was yielded through several rounds of real-space refinement by Phenix (54) and manual adjustment using Coot (55). In all three final models, the N-terminal residues Glu469–Phe471 and the C-terminal Trp509–Thr511 (or Ser510–Thr511) as well as 6xHis-tag of CHIKV-43 peptide cannot be built due to the absence of electron density. The complete NTF2Ls (residue 1–138) were built in four chains, C, G, N, and R, while other chains lack residues Met1–Glu4 owing to the incomplete electron density map.

All structural figures were generated using PyMOL (http://www.pymol.org), UCSF Chimera (56) (https://www.cgl.ucsf.edu/chimera/), or UCSF ChimeraX (57) (https://www.cgl.ucsf.edu/chimerax/).

### ITC

Freshly purified NTF2L protein was concentrated to 100 µM in HEPES buffer (20 mM HEPES, 150 mM NaCl, pH 7.5). Its ligand peptides (_473_ITFGDFNDGEIESLSSEL_490_ and _491_LTFGDFLPGEVDDLTDSD_508,_ synthesized by GL Biochem Ltd., Shanghai, China) were adjusted to 1 mM in the same buffer, respectively. In parallel, the CHIKV-43 peptide concentration was adjusted to 50 µM, and the corresponding NTF2L concentration was adjusted to 10 µM. The CHIKV-26 peptide concentration was adjusted to 500 µM, and the corresponding NTF2L concentration was adjusted to 50 µM. The CHIKV-43 mut peptide concentration was adjusted to 200 µM, and the corresponding NTF2L concentration was adjusted to 40 µM. The measurements of binding affinity were carried out using AFFINITY ITC (TA Instruments, USA). The titration assay was performed at 20°C by injecting 2.5 µL peptide into a full cell containing 182 µL NTF2L protein every 150 s–200 s for a total of 20 injections, and the stirrer syringe speed was set at 125 revolutions per minute. All raw titration data sets were processed in NanoAnalyze Data Analysis software, version 3.12.0.

### IF assay

HEK293T cells (National Collection of Authenticated Cell Cultures, Shanghai, China) were seeded into 24-well cell culture plates with glass coverslips and then transfected with the recombinant plasmids using TransIntro PEI Transfection Reagent (TransGen Biotech, FT401-01). Twelve hours after transfection, the cells were treated with 0.5 mM sodium arsenite for 1 h. The cells were washed three times with PBS buffer and then fixed in 4% paraformaldehyde for 20 min. Cells were permeabilized in PBS plus 0.3% Triton X-100 for 30 min and subsequently incubated in blocking solution (5% bovine serum albumin in PBS plus 0.1% Tween-20 [PBST]) for 4 h at 25°C. Samples were incubated with mouse anti-TIA-1 antibody (Santa Cruz, sc-166247) or mouse anti-G3BP1 antibody (Santa Cruz, sc-365338) overnight and incubated with Alexa 488-conjugated secondary antibody (Thermo, A-11029) and 4´,6-diamidino-2-phenylindole (Roche, 28718-90-3). After antibody incubations, samples were washed three times with PBST. Finally, coverslips were mounted on microscopy slides. Images were captured using ZEISS LSM 900 with Airyscan 2. The SG-positive cells were quantified from at least 50 cells per condition. All experiments were replicated three times.

### Real-time quantitative PCR (RT-qPCR)

HEK293T cells were seeded into 24-well cell culture plates and transfected with the recombinant plasmids using TransIntro EL Transfection Reagent (TransGen Biotech, FT201-01). Twelve hours after transfection, the cells were transfected with poly(I:C) (Sigma-Aldrich, P1530) for 12 h. Total RNA was isolated using the FastPure Cell/Tissue Total RNA Isolation Kit (Vazyme, RC101-01). Genomic DNA was then removed, and the reverse transcription was performed using Hifair III 1st Strand cDNA Synthesis SuperMix (YEASEN, 11141ES10) according to the manufacturer’s instruction. The expression levels of IFNβ and GAPDH were determined by RT-qPCR using a Hieff UNICON Universal Blue qPCR SYBR Green Master Mix (YEASEN, 11184ES03) and an Applied CFX96 Real-Time PCR Detection System (Bio-Rad). The sequences of the primers used in this assay are listed in Table S4. All experiments were replicated three times.

### Co-IP and western blot analysis

HEK293T cells were seeded into a six-well cell culture plate and then transfected with the recombinant plasmids using TransIntro PEI Transfection Reagent (TransGen Biotech, FT401-01). Thirty-six hours after transfection, the cells were lysed with cell lysis buffer (Beyotime, P0013J) supplemented with proteinase inhibitor phenylmethylsulfonyl fluoride (Solarbio, P8340). Cell lysates were then centrifuged, and the supernatants were immunoprecipitated with Flag magnetic beads (LABLEAD, PFM025). Beads were washed three times with washing buffer (50 mM Tris-HCl, 500 mM NaCl, 0.1% Triton X-100, 1 mM EDTA, pH 7.5). Cell lysates or immunoprecipitants were analyzed with immunoblotting.

The proteins were then transferred to polyvinylidene fluoride membranes. Membranes were blocked for 4 h with 5% non-fat dry milk solution in Tris-buffered saline containing 0.1% Tween-20 (TBST) and then incubated with primary antibodies mouse anti-G3BP1 (Santa Cruz, sc-365338), mouse anti-Flag (Proteintech, 66008-4-Ig), and rabbit anti-β-actin (Proteintech, 81115-1-RR) overnight at 4°C. The membranes were washed with TBST and then incubated with the corresponding secondary antibodies. Proteins were visualized by SuperKine West Femto Maximum Sensitivity Substrate according to the manufacturer’s instruction (Abbkine, BMU102).

### Quantification and statistical analysis

Statistical analysis was performed in GraphPad Prism 8 Software. Differences between the two groups were analyzed using Student’s *t*-test and considered significant at *P < 0.05, **P < 0.01, ***P < 0.001, and ****P < 0.0001. *P* > 0.05 was considered not significant. All data are presented as mean ± standard deviation.

## Data Availability

The data in this study are available in the main text and the supplemental material. The coordinates of the models have been deposited into the Protein Data Bank (PDB) under accession codes 9IVQ (Conformation I), 9IVR (Conformation II), and 9IVS (Conformation III). The cryo-EM maps have been deposited to the Electron Microscopy Data Bank (EMDB) under accession numbers EMD-60932 (Conformation I), EMD-60933 (Conformation II), and EMD-60934 (Conformation III).

## AFTER EPUB

*[This article was published on 11 April 2025 with an error in Acknowledgments. The error was corrected in the current version, posted on 15 April 2025.]*

## SUPPLEMENTAL MATERIAL

Legend mbio.03967-24-s0001.docx Video S1 legend.

Supplemental Material mbio.03967-24-s0002.docx Fig. S1 to S8; Tables S1 to S4.

Video S1 mbio.03967-24-s0003.mp4 Dynamic transformations among the three conformations of the CHIKV-43–NTF2L complex in solution.

ASM does not own the copyrights to Supplemental Material that may be linked to, or accessed through, an article. The authors have granted ASM a non-exclusive, world-wide license to publish the Supplemental Material files. Please contact the corresponding author directly for reuse.

## References

[1] Kumar R, Ahmed S, Parray HA, Das S. 2021. Chikungunya and arthritis: an overview. Travel Med Infect Dis 44:102168. doi:10.1016/j.tmaid.2021.102168 34563686

[2] Khongwichit S, Chansaenroj J, Chirathaworn C, Poovorawan Y. 2021. Chikungunya virus infection: molecular biology, clinical characteristics, and epidemiology in Asian countries. J Biomed Sci 28:84. doi:10.1186/s12929-021-00778-8 34857000 PMC8638460

[3] Tritsch SR, Encinales L, Pacheco N, Cadena A, Cure C, McMahon E, Watson H, Porras Ramirez A, Mendoza AR, Li G, et al.. 2020. Chronic joint pain 3 years after chikungunya virus infection largely characterized by relapsing-remitting symptoms. J Rheumatol 47:1267–1274. doi:10.3899/jrheum.190162 31263071 PMC7938419

[4] Evans-Gilbert T. 2017. Chikungunya and neonatal immunity: fatal vertically transmitted chikungunya infection. Am J Trop Med Hyg 96:913–915. doi:10.4269/ajtmh.16-0491 28167590 PMC5392641

[5] Dorléans F, Hoen B, Najioullah F, Herrmann-Storck C, Schepers KM, Abel S, Lamaury I, Fagour L, Césaire R, Guyomard S, et al.. 2018. Outbreak of Chikungunya in the French Caribbean islands of martinique and guadeloupe: findings from a hospital-based surveillance system (2013-2015). Am J Trop Med Hyg 98:1819–1825. doi:10.4269/ajtmh.16-0719 29692295 PMC6086161

[6] de Souza WM, Fumagalli MJ, de Lima STS, Parise PL, Carvalho DCM, Hernandez C, de Jesus R, Delafiori J, Candido DS, Carregari VC, et al.. 2024. Pathophysiology of chikungunya virus infection associated with fatal outcomes. Cell Host Microbe 32:606–622. doi:10.1016/j.chom.2024.02.011 38479396 PMC11018361

[7] Hua C, Combe B. 2017. Chikungunya virus-associated disease. Curr Rheumatol Rep 19:69. doi:10.1007/s11926-017-0694-0 28983760

[8] Silva MMO, Tauro LB, Kikuti M, Anjos RO, Santos VC, Gonçalves TSF, Paploski IAD, Moreira PSS, Nascimento LCJ, Campos GS, Ko AI, Weaver SC, Reis MG, Kitron U, Ribeiro GS. 2019. Concomitant transmission of dengue, chikungunya, and Zika viruses in brazil: clinical and epidemiological findings from surveillance for acute febrile illness. Clin Infect Dis 69:1353–1359. doi:10.1093/cid/ciy1083 30561554 PMC7348233

[9] Ly H. 2024. Ixchiq (VLA1553): the first FDA-approved vaccine to prevent disease caused by Chikungunya virus infection. Virulence 15:2301573. doi:10.1080/21505594.2023.2301573 38217381 PMC10793683

[10] Schneider M, Narciso-Abraham M, Hadl S, McMahon R, Toepfer S, Fuchs U, Hochreiter R, Bitzer A, Kosulin K, Larcher-Senn J, Mader R, Dubischar K, Zoihsl O, Jaramillo JC, Eder-Lingelbach S, Buerger V, Wressnigg N. 2023. Safety and immunogenicity of a single-shot live-attenuated chikungunya vaccine: a double-blind, multicentre, randomised, placebo-controlled, phase 3 trial. The Lancet 401:2138–2147. doi:10.1016/S0140-6736(23)00641-4 PMC10314240 37321235

[11] Ng LFP, Rénia L. 2024. Live-attenuated chikungunya virus vaccine. Cell 187:813–813. doi:10.1016/j.cell.2024.01.033 38364787

[12] Tan YB, Chmielewski D, Law MCY, Zhang K, He Y, Chen M, Jin J, Luo D. 2022. Molecular architecture of the Chikungunya virus replication complex. Sci Adv 8:eadd2536. doi:10.1126/sciadv.add2536 36449616 PMC9710867

[13] Laurent T, Kumar P, Liese S, Zare F, Jonasson M, Carlson A, Carlson LA. 2022. Architecture of the chikungunya virus replication organelle. Elife 11:e83042. doi:10.7554/eLife.83042 36259931 PMC9633065

[14] Girard J, Le-Bihan O, Lai-Kee-Him J, Girleanu M, Bernard E, Castellarin C, Neyret A, Spehner D, Holy X, Favier A-L, Briant L, Bron P. 2023. In situ characterization of late Chikungunya virus replication organelle. BioRxiv. doi:10.1101/2023.02.25.530016 PMC11265437 38940586

[15] Yap ML, Klose T, Urakami A, Hasan SS, Akahata W, Rossmann MG. 2017. Structural studies of Chikungunya virus maturation. Proc Natl Acad Sci USA 114:13703–13707. doi:10.1073/pnas.1713166114 29203665 PMC5748190

[16] Götte B, Liu L, McInerney GM. 2018. The enigmatic alphavirus non-structural protein 3 (nsP3) revealing its secrets at last. Viruses 10:105. doi:10.3390/v10030105 29495654 PMC5869498

[17] McPherson RL, Abraham R, Sreekumar E, Ong S-E, Cheng S-J, Baxter VK, Kistemaker HAV, Filippov DV, Griffin DE, Leung AKL. 2017. ADP-ribosylhydrolase activity of Chikungunya virus macrodomain is critical for virus replication and virulence. Proc Natl Acad Sci USA 114:1666–1671. doi:10.1073/pnas.1621485114 28143925 PMC5321000

[18] Krieg S, Pott F, Potthoff L, Verheirstraeten M, Bütepage M, Golzmann A, Lippok B, Goffinet C, Lüscher B, Korn P. 2023. Mono-ADP-ribosylation by PARP10 inhibits Chikungunya virus nsP2 proteolytic activity and viral replication. Cell Mol Life Sci 80:72. doi:10.1007/s00018-023-04717-8 36840772 PMC9959937

[19] Gao Y, Goonawardane N, Ward J, Tuplin A, Harris M. 2019. Multiple roles of the non-structural protein 3 (nsP3) alphavirus unique domain (AUD) during Chikungunya virus genome replication and transcription. PLoS Pathog 15:e1007239. doi:10.1371/journal.ppat.1007239 30668592 PMC6358111

[20] Kril V, Hons M, Amadori C, Zimberger C, Couture L, Bouery Y, Burlaud-Gaillard J, Karpov A, Ptchelkine D, Thienel AL, Kümmerer BM, Desfosses A, Jones R, Roingeard P, Meertens L, Amara A, Reguera J. 2024. Alphavirus nsP3 organizes into tubular scaffolds essential for infection and the cytoplasmic granule architecture. Nat Commun 15:8106. doi:10.1038/s41467-024-51952-z 39285216 PMC11405681

[21] Meshram CD, Agback P, Shiliaev N, Urakova N, Mobley JA, Agback T, Frolova EI, Frolov I. 2018. Multiple host factors interact with the hypervariable domain of Chikungunya virus nsP3 and determine viral replication in cell-specific mode. J Virol 92:e00838-18. doi:10.1128/JVI.00838-18 29899097 PMC6069204

[22] Tossavainen H, Aitio O, Hellman M, Saksela K, Permi P. 2016. Structural basis of the high affinity interaction between the alphavirus nonstructural protein-3 (nsP3) and the SH3 domain of amphiphysin-2. J Biol Chem 291:16307–16317. doi:10.1074/jbc.M116.732412 27268056 PMC4965578

[23] Meertens L, Hafirassou ML, Couderc T, Bonnet-Madin L, Kril V, Kümmerer BM, Labeau A, Brugier A, Simon-Loriere E, Burlaud-Gaillard J, et al.. 2019. FHL1 is a major host factor for chikungunya virus infection. Nature 574:259–263. doi:10.1038/s41586-019-1578-4 31554973

[24] Panas MD, Schulte T, Thaa B, Sandalova T, Kedersha N, Achour A, McInerney GM. 2015. Viral and cellular proteins containing FGDF motifs bind G3BP to block stress granule formation. PLoS Pathog 11:e1004659. doi:10.1371/journal.ppat.1004659 25658430 PMC4450067

[25] Kristensen O. 2015. Crystal structure of the G3BP2 NTF2-like domain in complex with a canonical FGDF motif peptide. Biochem Biophys Res Commun 467:53–57. doi:10.1016/j.bbrc.2015.09.123 26410532

[26] Schulte T, Liu L, Panas MD, Thaa B, Dickson N, Götte B, Achour A, McInerney GM. 2016. Combined structural, biochemical and cellular evidence demonstrates that both FGDF motifs in alphavirus nsP3 are required for efficient replication. Open Biol 6:160078. doi:10.1098/rsob.160078 27383630 PMC4967826

[27] Panas MD, Ahola T, McInerney GM. 2014. The C-terminal repeat domains of nsP3 from the Old World alphaviruses bind directly to G3BP. J Virol 88:5888–5893. doi:10.1128/JVI.00439-14 24623412 PMC4019107

[28] Yang P, Mathieu C, Kolaitis RM, Zhang P, Messing J, Yurtsever U, Yang Z, Wu J, Li Y, Pan Q, Yu J, Martin EW, Mittag T, Kim HJ, Taylor JP. 2020. G3BP1 is a tunable switch that triggers phase separation to assemble stress granules. Cell 181:325–345. doi:10.1016/j.cell.2020.03.046 32302571 PMC7448383

[29] Kang JS, Hwang YS, Kim LK, Lee S, Lee WB, Kim-Ha J, Kim YJ. 2018. OASL1 traps viral rnas in stress granules to promote antiviral responses. Mol Cells 41:214–223. doi:10.14348/molcells.2018.2293 29463066 PMC5881095

[30] McCormick C, Khaperskyy DA. 2017. Translation inhibition and stress granules in the antiviral immune response. Nat Rev Immunol 17:647–660. doi:10.1038/nri.2017.63 28669985

[31] Kim SS-Y, Sze L, Liu C, Lam K-P. 2019. The stress granule protein G3BP1 binds viral dsRNA and RIG-I to enhance interferon-β response. J Biol Chem 294:6430–6438. doi:10.1074/jbc.RA118.005868 30804210 PMC6484135

[32] Liu ZS, Cai H, Xue W, Wang M, Xia T, Li WJ, Xing JQ, Zhao M, Huang YJ, Chen S, Wu SM, Wang X, Liu X, Pang X, Zhang ZY, Li T, Dai J, Dong F, Xia Q, Li AL, Zhou T, Liu ZG, Zhang XM, Li T. 2019. G3BP1 promotes DNA binding and activation of cGAS. Nat Immunol 20:18–28. doi:10.1038/s41590-018-0262-4 30510222 PMC8276115

[33] Yang W, Ru Y, Ren J, Bai J, Wei J, Fu S, Liu X, Li D, Zheng H. 2019. G3BP1 inhibits RNA virus replication by positively regulating RIG-I-mediated cellular antiviral response. Cell Death Dis 10:946. doi:10.1038/s41419-019-2178-9 31827077 PMC6906297

[34] Zhao M, Xia T, Xing JQ, Yin LH, Li XW, Pan J, Liu JY, Sun LM, Wang M, Li T, Mao J, Han QY, Xue W, Cai H, Wang K, Xu X, Li T, He K, Wang N, Li AL, Zhou T, Zhang XM, Li WH, Li T. 2022. The stress granule protein G3BP1 promotes pre‐condensation of cGAS to allow rapid responses to DNA. EMBO Reports 23. doi:10.15252/embr.202153166 PMC8728604 34779554

[35] White JP, Cardenas AM, Marissen WE, Lloyd RE. 2007. Inhibition of cytoplasmic mRNA stress granule formation by a viral proteinase. Cell Host Microbe 2:295–305. doi:10.1016/j.chom.2007.08.006 18005751

[36] Visser LJ, Medina GN, Rabouw HH, de Groot RJ, Langereis MA, de Los Santos T, van Kuppeveld FJM. 2019. Foot-and-mouth disease virus leader protease cleaves G3BP1 and G3BP2 and inhibits stress granule formation. J Virol 93:e00922-18. doi:10.1128/JVI.00922-18 30404792 PMC6321903

[37] Zheng Y, Deng J, Han L, Zhuang MW, Xu Y, Zhang J, Nan ML, Xiao Y, Zhan P, Liu X, Gao C, Wang PH. 2022. SARS-CoV-2 NSP5 and N protein counteract the RIG-I signaling pathway by suppressing the formation of stress granules. Signal Transduct Target Ther 7:22. doi:10.1038/s41392-022-00878-3 35075101 PMC8785035

[38] Biswal M, Lu J, Song J. 2022. SARS-CoV-2 nucleocapsid protein targets a conserved surface groove of the NTF2-like domain of G3BP1. J Mol Biol 434:167516. doi:10.1016/j.jmb.2022.167516 35240128 PMC8882607

[39] Fros JJ, Domeradzka NE, Baggen J, Geertsema C, Flipse J, Vlak JM, Pijlman GP. 2012. Chikungunya virus nsP3 blocks stress granule assembly by recruitment of G3BP into cytoplasmic foci. J Virol 86:10873–10879. doi:10.1128/JVI.01506-12 22837213 PMC3457282

[40] Panas MD, Varjak M, Lulla A, Eng KE, Merits A, Karlsson Hedestam GB, McInerney GM. 2012. Sequestration of G3BP coupled with efficient translation inhibits stress granules in Semliki Forest virus infection. MBoC 23:4701–4712. doi:10.1091/mbc.e12-08-0619 23087212 PMC3521679

[41] Kim DY, Reynaud JM, Rasalouskaya A, Akhrymuk I, Mobley JA, Frolov I, Frolova EI. 2016. New world and old world alphaviruses have evolved to exploit different components of stress granules, FXR and G3BP proteins, for assembly of viral replication complexes. PLoS Pathog 12:e1005810. doi:10.1371/journal.ppat.1005810 27509095 PMC4980055

[42] Song D, Kuang L, Yang L, Wang L, Li H, Li X, Zhu Z, Shi C, Zhu H, Gong W. 2022. Yin and yang regulation of stress granules by Caprin-1. Proc Natl Acad Sci USA 119:e2207975119. doi:10.1073/pnas.2207975119 36279435 PMC9636964

[43] Vognsen T, Møller IR, Kristensen O. 2013. Crystal structures of the human G3BP1 NTF2-like domain visualize FxFG Nup repeat specificity. PLoS One 8:e80947. doi:10.1371/journal.pone.0080947 24324649 PMC3852005

[44] Nowee G, Bakker JW, Geertsema C, Ros VID, Göertz GP, Fros JJ, Pijlman GP. 2021. A tale of 20 alphaviruses; inter-species diversity and conserved interactions between viral non-structural protein 3 and stress granule proteins. Front Cell Dev Biol 9:625711. doi:10.3389/fcell.2021.625711 33644063 PMC7905232

[45] Jayabalan AK, Adivarahan S, Koppula A, Abraham R, Batish M, Zenklusen D, Griffin DE, Leung AKL. 2021. Stress granule formation, disassembly, and composition are regulated by alphavirus ADP-ribosylhydrolase activity. Proc Natl Acad Sci USA 118:e2021719118. doi:10.1073/pnas.2021719118 33547245 PMC8017970

[46] Hou S, Kumar A, Xu Z, Airo AM, Stryapunina I, Wong CP, Branton W, Tchesnokov E, Götte M, Power C, Hobman TC. 2017. Zika virus hijacks stress granule proteins and modulates the host stress response. J Virol 91:e00474-17. doi:10.1128/JVI.00474-17 28592527 PMC5533921

[47] Li T, Zeng Z, Fan C, Xiong W. 2023. Role of stress granules in tumorigenesis and cancer therapy. Biochim Biophys Acta Rev Cancer 1878:189006. doi:10.1016/j.bbcan.2023.189006 37913942

[48] Zhan Y, Wang H, Ning Y, Zheng H, Liu S, Yang Y, Zhou M, Fan S. 2020. Understanding the roles of stress granule during chemotherapy for patients with malignant tumors. Am J Cancer Res 10:2226–2241. 32905441 PMC7471355

[49] Wolozin B, Ivanov P. 2019. Stress granules and neurodegeneration. Nat Rev Neurosci 20:649–666. doi:10.1038/s41583-019-0222-5 31582840 PMC6986315

[50] Ali N, Prasad K, AlAsmari AF, Alharbi M, Rashid S, Kumar V. 2021. Genomics-guided targeting of stress granule proteins G3BP1/2 to inhibit SARS-CoV-2 propagation. Int J Biol Macromol 190:636–648. doi:10.1016/j.ijbiomac.2021.09.018 34517025 PMC8431879

[51] Zhang H, Zhang S, He H, Zhao W, Chen J, Shao R. 2012. GAP161 targets and downregulates G3BP to suppress cell growth and potentiate cisplaitin-mediated cytotoxicity to colon carcinoma HCT116 cells. Cancer Sci 103:1848–1856. doi:10.1111/j.1349-7006.2012.02361.x 22703643 PMC7659306

[52] Shim JH, Su ZY, Chae JI, Kim DJ, Zhu F, Ma WY, Bode AM, Yang CS, Dong Z. 2010. Epigallocatechin gallate suppresses lung cancer cell growth through Ras–GTPase-activating protein SH3 domain-binding protein 1. Cancer Prev Res (Phila) 3:670–679. doi:10.1158/1940-6207.CAPR-09-0185 20424128

[53] Punjani A, Rubinstein JL, Fleet DJ, Brubaker MA. 2017. cryoSPARC: algorithms for rapid unsupervised cryo-EM structure determination. Nat Methods 14:290–296. doi:10.1038/nmeth.4169 28165473

[54] Adams PD, Afonine PV, Bunkóczi G, Chen VB, Davis IW, Echols N, Headd JJ, Hung LW, Kapral GJ, Grosse-Kunstleve RW, McCoy AJ, Moriarty NW, Oeffner R, Read RJ, Richardson DC, Richardson JS, Terwilliger TC, Zwart PH. 2010. PHENIX: a comprehensive python-based system for macromolecular structure solution. Acta Crystallogr D Biol Crystallogr 66:213–221. doi:10.1107/S0907444909052925 20124702 PMC2815670

[55] Emsley P, Lohkamp B, Scott WG, Cowtan K. 2010. Features and development of coot. Acta Crystallogr D Biol Crystallogr 66:486–501. doi:10.1107/S0907444910007493 20383002 PMC2852313

[56] Pettersen EF, Goddard TD, Huang CC, Couch GS, Greenblatt DM, Meng EC, Ferrin TE. 2004. UCSF Chimera—a visualization system for exploratory research and analysis. J Comput Chem 25:1605–1612. doi:10.1002/jcc.20084 15264254

[57] Goddard TD, Huang CC, Meng EC, Pettersen EF, Couch GS, Morris JH, Ferrin TE. 2018. UCSF ChimeraX: meeting modern challenges in visualization and analysis. Protein Sci 27:14–25. doi:10.1002/pro.3235 28710774 PMC5734306

