# Supplementary material for: Cryo-EM reveals a double oligomeric ring scaffold of the CHIKV nsP3 peptide in complex with the NTF2L domain of host G3BP1
Source: mBio. 2025 Apr 11;16(5):e03967-24. doi: 10.1128/mbio.03967-24 (PMC12077208; doi:10.1128/mbio.03967-24)
Supplement: Legend — Video S1 legend. [file mbio.03967-24-s0001.docx]

**Video S1. (separate file)**

Dynamic transformations among the three conformations of the CHIKV-43–NTF2L complex in solution.
