# Supplementary material for: Cryo-EM reveals a double oligomeric ring scaffold of the CHIKV nsP3 peptide in complex with the NTF2L domain of host G3BP1
Source: mBio. 2025 Apr 11;16(5):e03967-24. doi: 10.1128/mbio.03967-24 (PMC12077208; doi:10.1128/mbio.03967-24)
Supplement: Supplemental Material — Fig. S1 to S8; Tables S1 to S4. [file mbio.03967-24-s0002.docx]

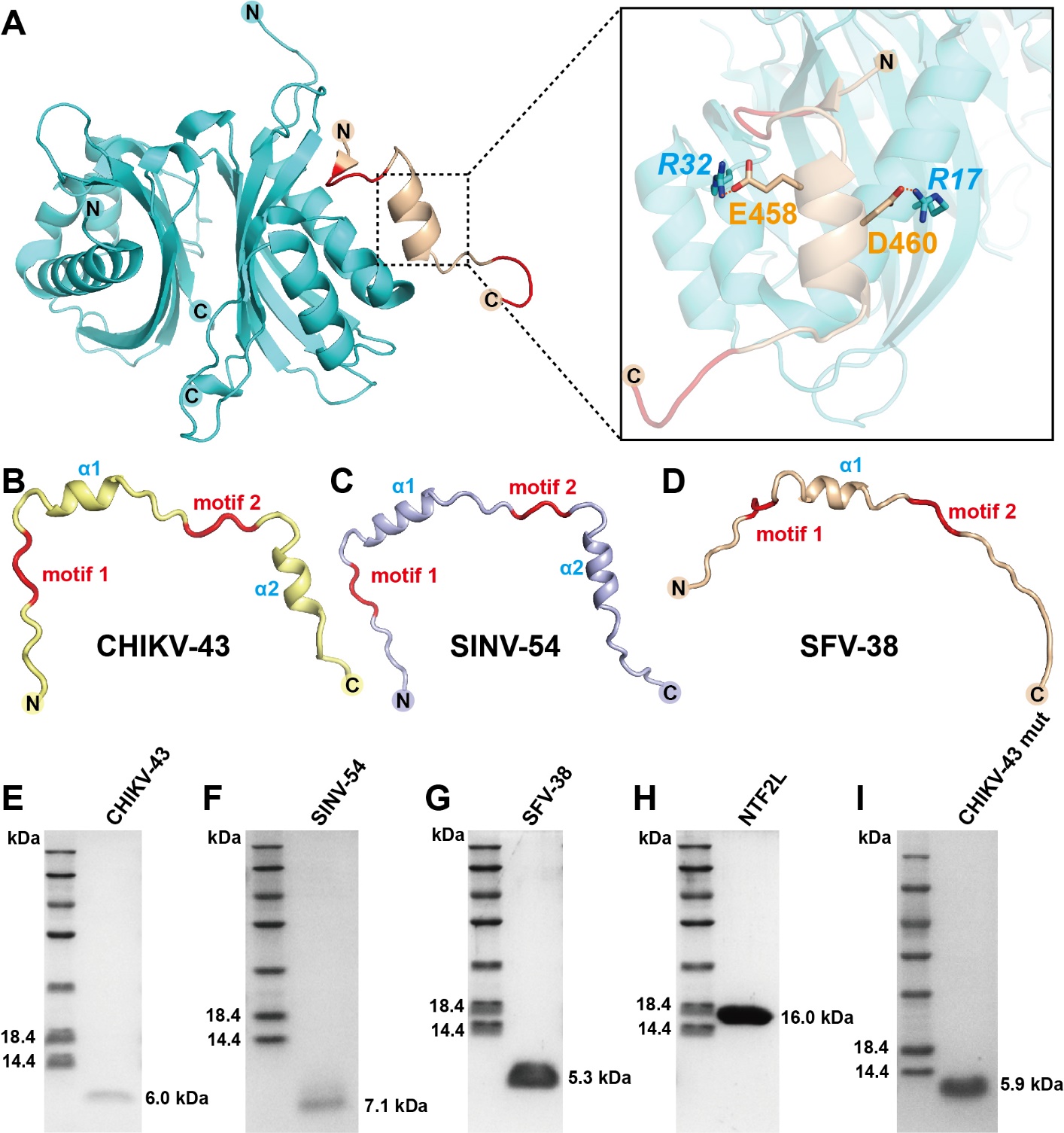


**Figure S1. Preparation of CHIKV-43, SINV-54, SFV-38, and the NTF2L domain of G3BP1.**

(A) The crystal structure of the SFV nsP3-25 (residues 449–473) in complex with the NTF2L domain (PDB code: 5FW5, (1)). The NTF2L dimer and SFV nsP3-25 are marked in cyan and wheat, respectively. Two FGDF motifs are colored in red. Residues Glu458 and Asp460 of the helix (α1) in the SFV nsP3-25 peptide form ionic interactions with *Arg32* and *Arg17* in NTF2L, respectively. (B–D) The structural models of CHIKV-43 (B), SINV-54 (C), and SFV-38 (D). Two FGDF motifs are labelled in red. These models were predicted using AlphaFold 3 (https://alphafoldserver.com/). (E–I) SDS-PAGE analysis of the purified CHIKV-43 (E), SINV-54 (F), SFV-38 (G), NTF2L (H), and CHIKV-43 mut (I). Figures (A–D) were prepared using PyMOL (http://www.pymol.org).


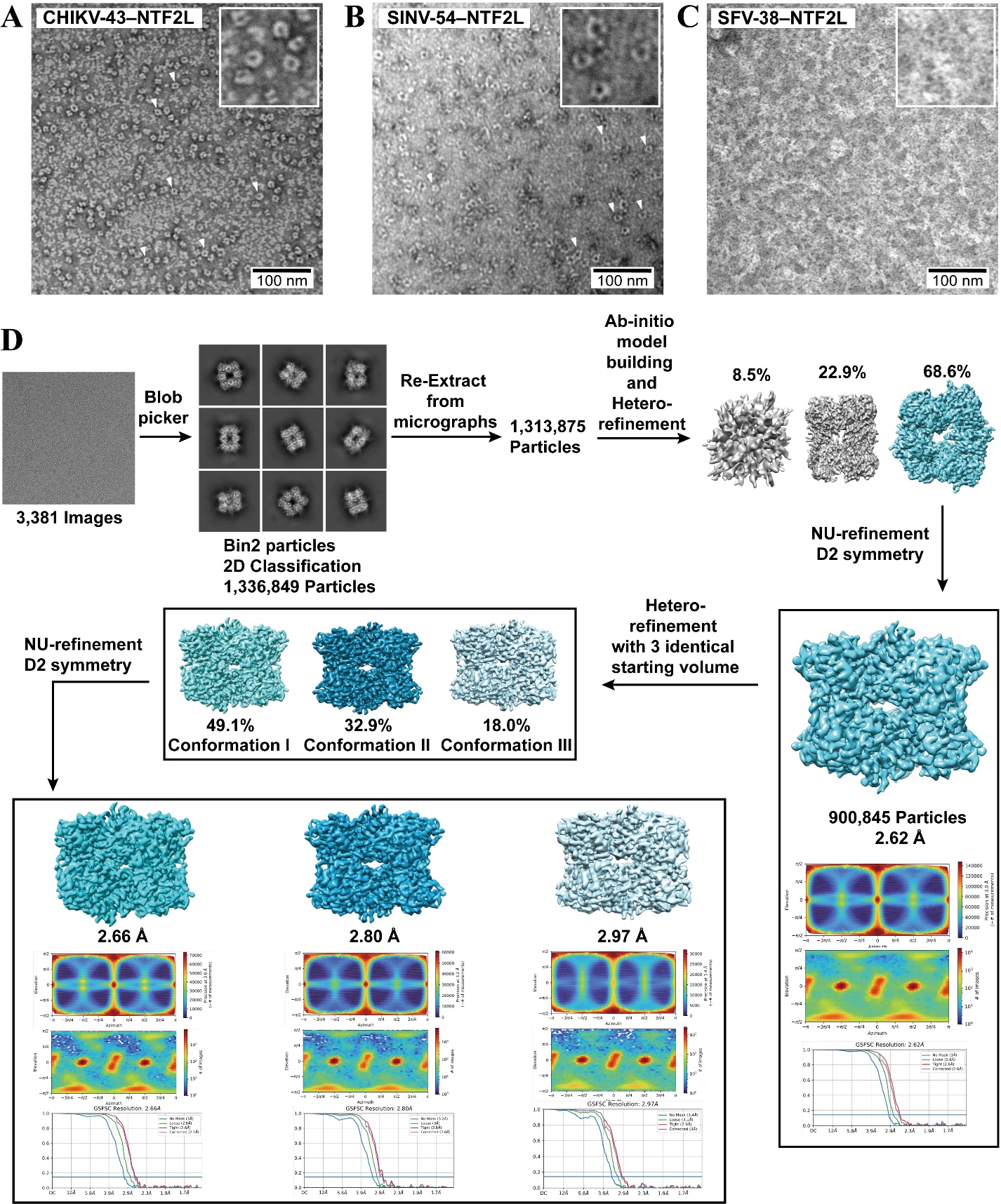


**Figure S2. Negative-staining electron microscopy and structure determination of the CHIKV-43–NTF2L complex.**

(A–C) Negative-staining images of the CHIKV-43–NTF2L (A), SINV-54–NTF2L (B), and SFV-38–NTF2L (C) complexes. (D) Workflow of the 3D reconstruction of the CHIKV-43–NTF2L complex.


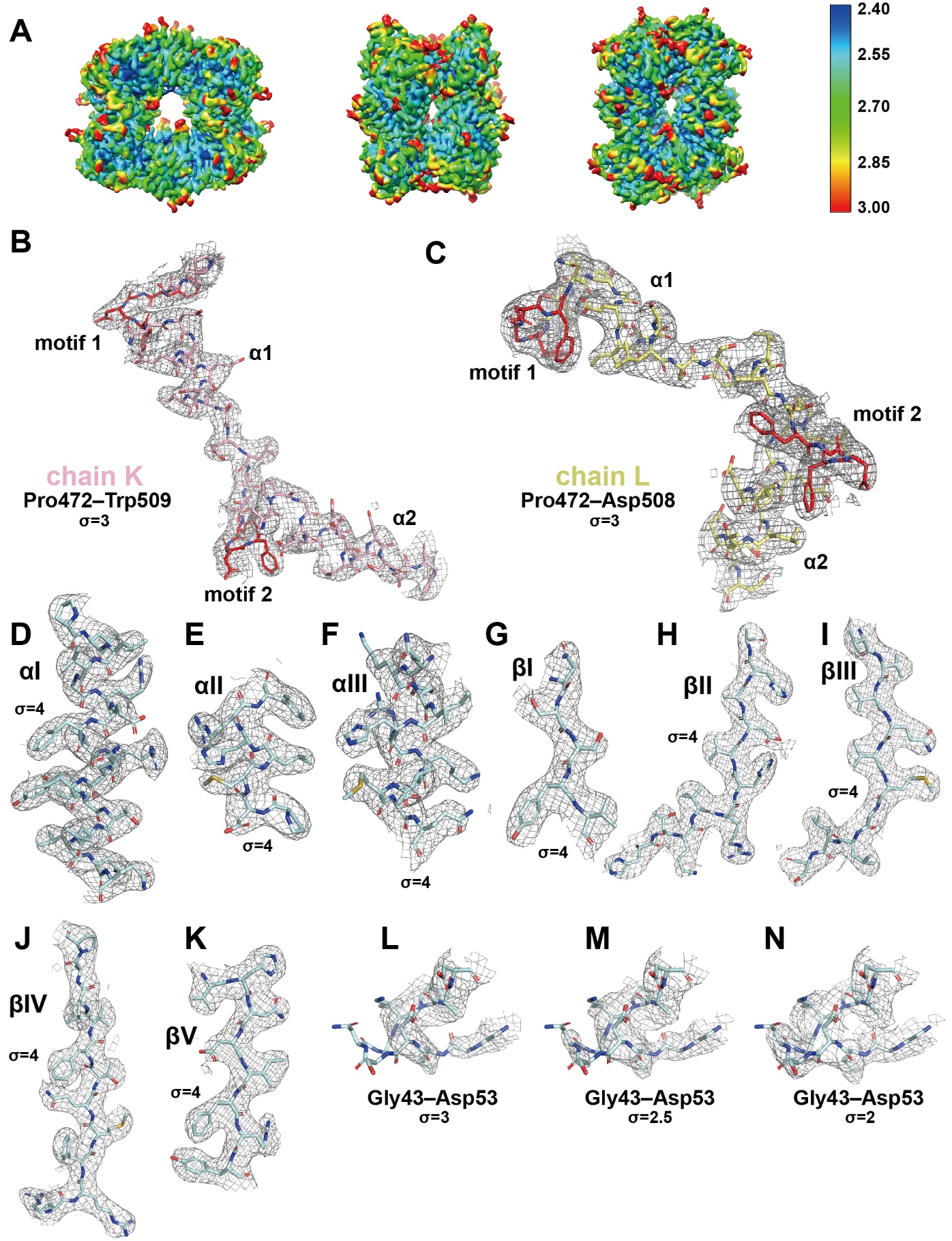


**Figure S3. Local resolution map and details of the electron density maps.**

(A) The reconstruction colored according to the estimated local resolution. Resolution is lower at the C-terminus of CHIKV-43 (Trp509–Thr511) and in a loop of NTF2L (Gly43–Asp53). (B and C) Electron density maps of two distinct CHIKV-43 conformations (using chain K and L as examples). Two FGDF motifs are colored in red. (D–K) Electron density maps of three α-helices (αI-III) and five β-strands (βI-V) of the NTF2L domain. (L–N) The electron density map of the loop (Gly43–Asp53) in the NTF2L domain, which are displayed at three decreasing contour levels. Electron density maps are displayed in gray mesh at the indicated σ. Figure (A) was prepared using UCSF Chimera (2) (https://www.cgl.ucsf.edu/chimera/) and figures (B–N) were generated using PyMOL (http://www.pymol.org).


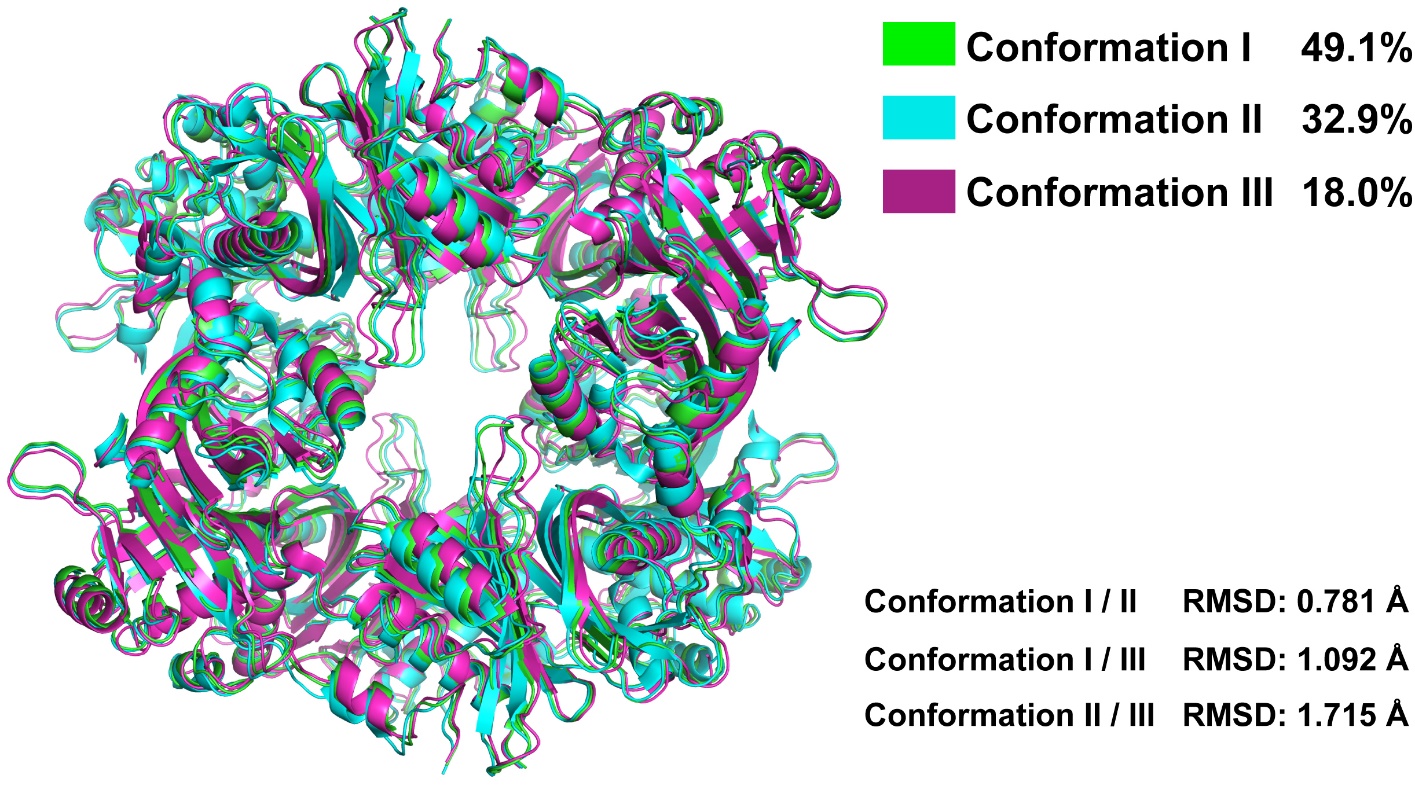


**Figure S4. Superimposition of three conformations of the CHIKV-43–NTF2L complex.**

Conformation I, II, and III are colored in green, cyan, and magenta, respectively. The figure was generated using PyMOL (http://www.pymol.org).


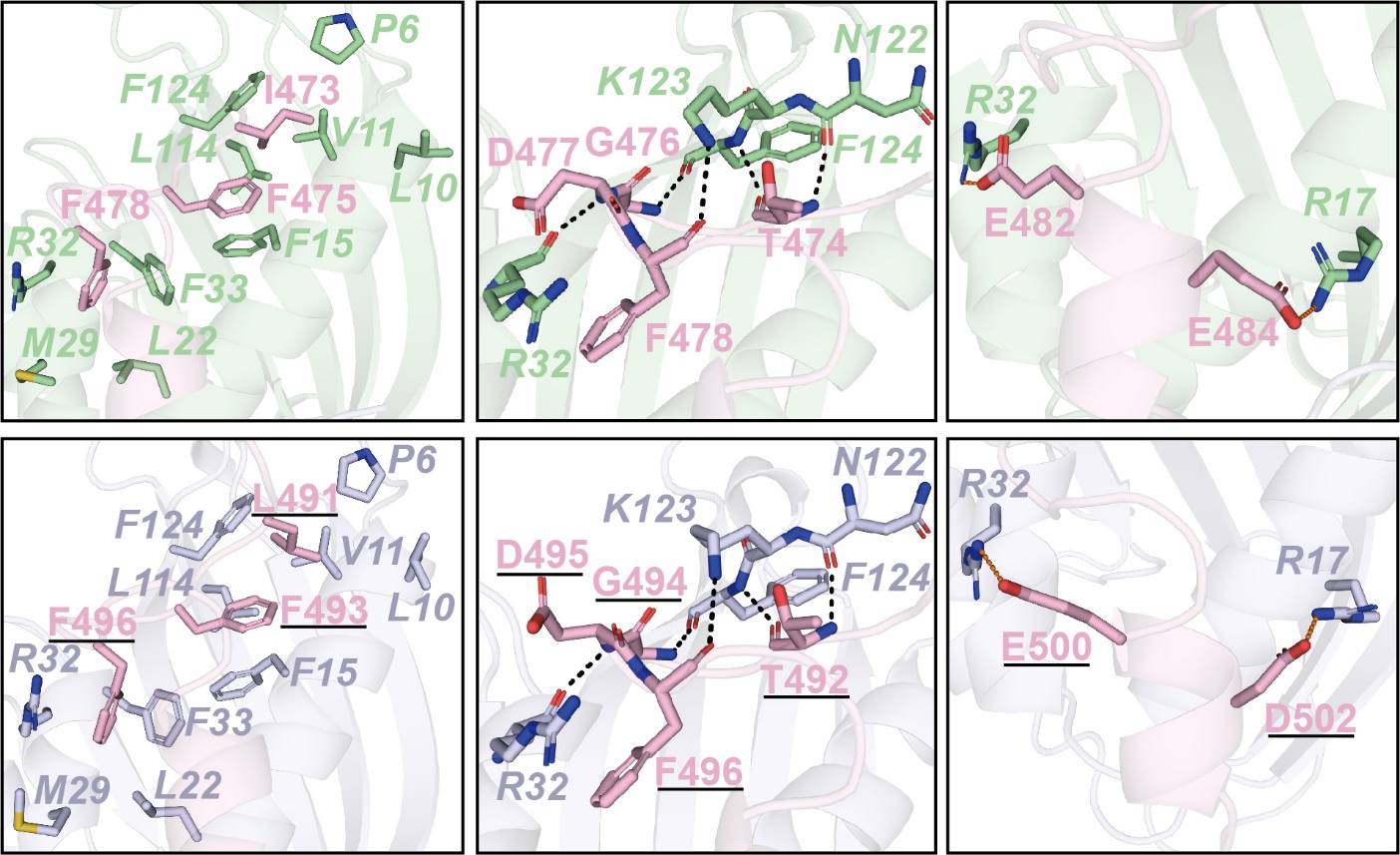


**Figure S5. Detailed interactions between CHIKV-43 and NTF2L in the extended conformation.**

Hydrogen (H)-bonds are showed by black dashed lines. Ionic bonds are presented by red dashed lines. These figures were generated using PyMOL (http://www.pymol.org).


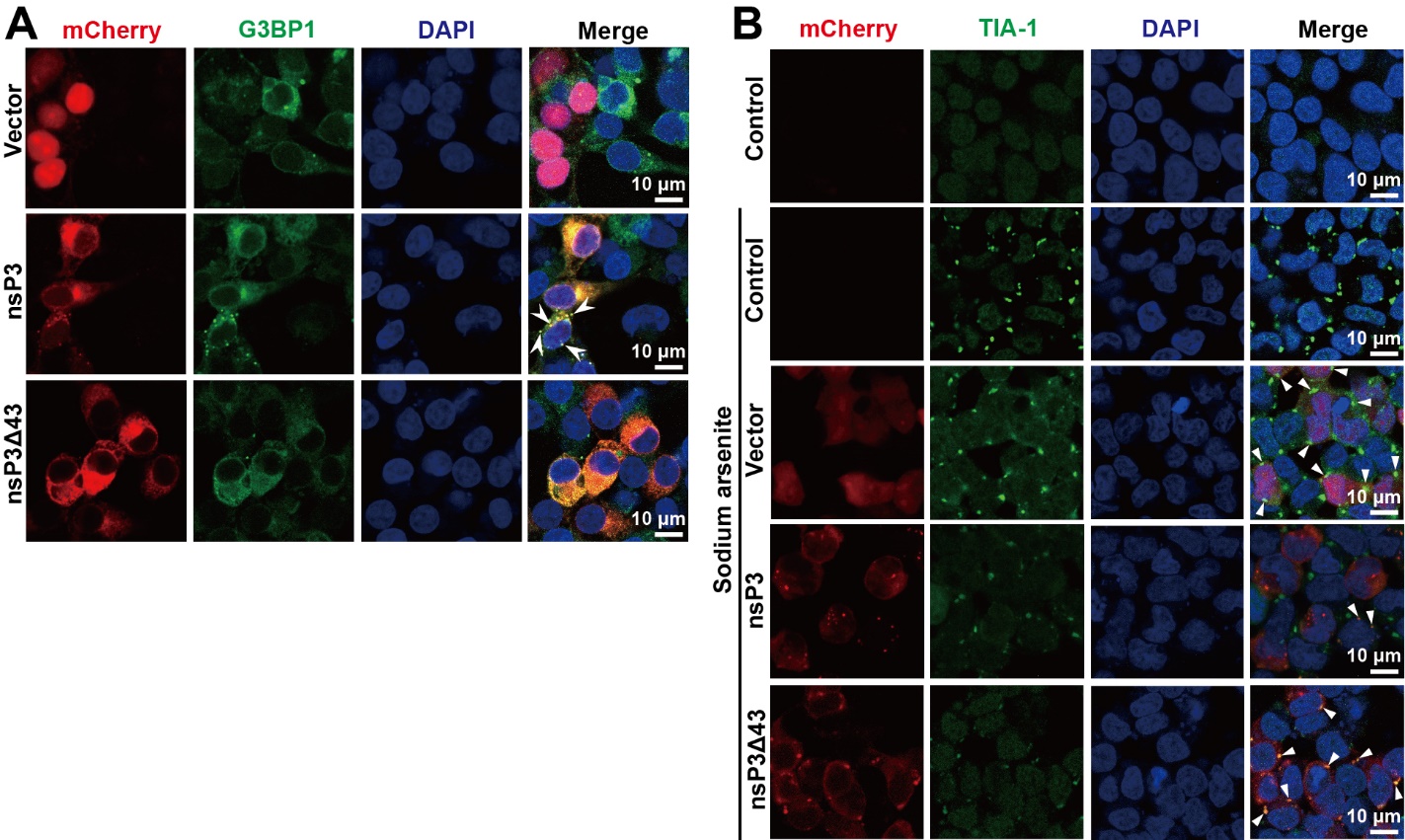


**Figure S6. The impact of CHIKV-43 on nsP3-G3BP1 colocalization and SG formation.**

(A) Colocalization between nsP3, nsP3Δ43, and endogenous G3BP1 in HEK293T cells. White arrows indicate G3BP1/nsP3 foci. Scale bar, 10 μm. (B) The impact of CHIKV nsP3, nsP3Δ43 on SG formation. At 12 h post transfection, HEK293T cells were stressed with 0.5 mM sodium arsenite for 1 h. Subsequently, immunofluorescence staining was performed for TIA-1. SGs are indicated by white-triangle arrows. Scale bar, 10 μm.


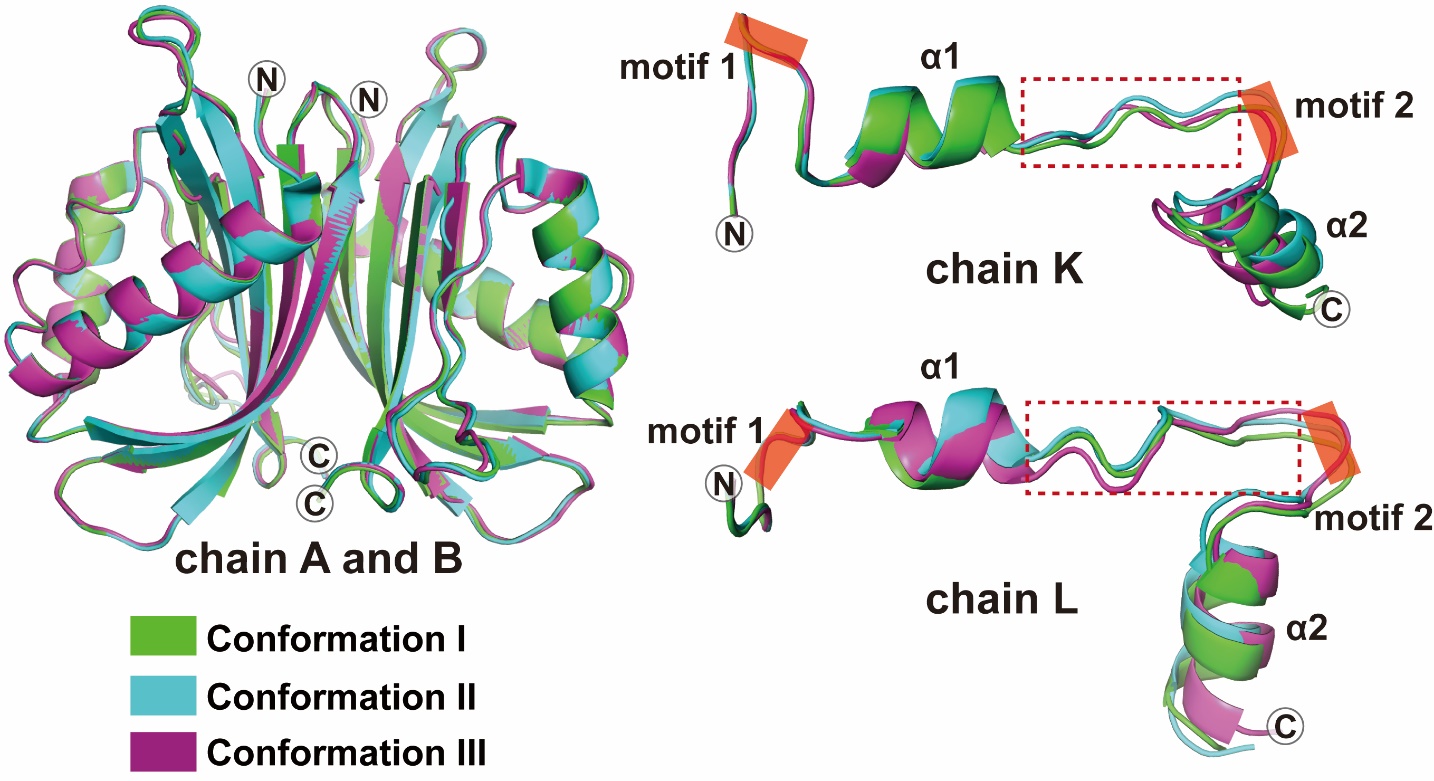


**Figure S7.** **The possible reason on the conformational changes of the CHIKV-43–NTF2L complex.**

The structures of NTF2Ls are consistent in three conformations (e.g., chains A and B). The flexible loops (indicated by the red dashed boxes) between α1 and motif 2 in the CHIKV-43 peptides exhibit conformational variability. Two FGDF motifs are marked with red boxes. These figures were generated using PyMOL (http://www.pymol.org).


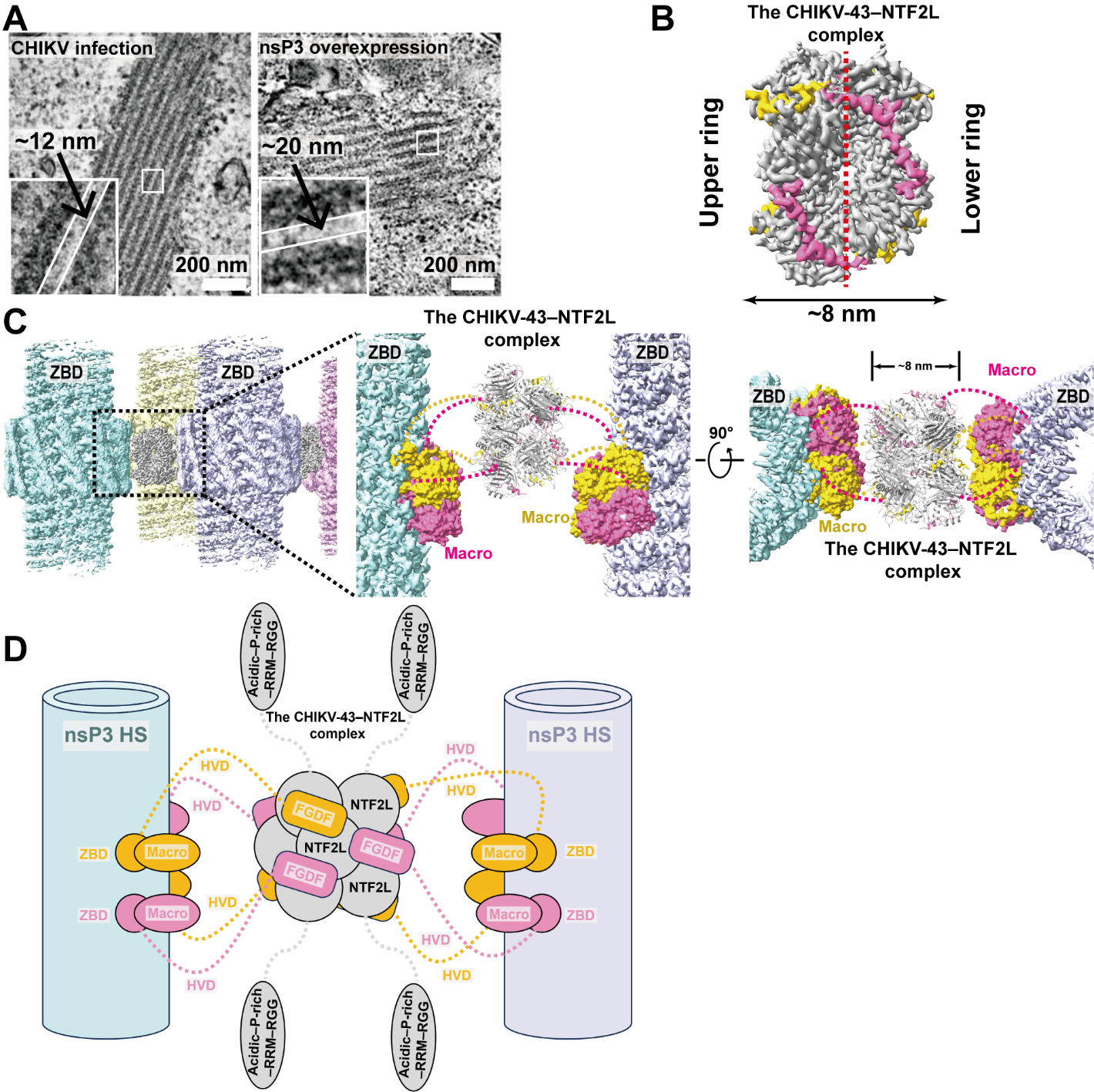


**Figure S8.** **The proposed interaction mode between full-length CHIKV nsP3 and host G3BP1.**

(A) Transmission electron microscopy of human fibroblasts infected with CHIKV or stably expressing viral nsP3 (3). Scale bar, 200 nm. (B) The width of cryo-EM CHIKV-43–NTF2L structure (Conformation I). (C) The structural model of the interaction between CHIKV nsP3 and G3BP1. The double-layered structure of CHIKV-43–NTF2L complex may connect adjacent nsP3 HSs, facilitating the formation of the higher-order tubular structures. The CHIKV-43 peptides in the upper ring are derived from the four nsP3s of one HS, while those in the lower ring originate from the four nsP3s of a different HS. The cryo-EM map of nsP3 HS was obtained from the Electron Microscopy Data Bank (EMDB code: EMD-17729 and EMD-17730). To distinguish two different conformations of the CHIKV-43 peptide in HVD region, CHIKV nsP3s are labeled in pink and yellow, respectively. NTF2L and other domains of G3BP1 are presented in gray. (D) Schematic diagram of the interaction mode between CHIKV nsP3 and G3BP1, displayed in the same colors as figure (C). Figures B and C were generated using UCSF ChimeraX (4) (https://www.cgl.ucsf.edu/chimerax/).

**Table S1. Cryo-EM data collection, data processing, and model building statistics.**

|  | **The CHIKV-43**–**NTF2L complex** | | |
| --- | --- | --- | --- |
|  | **Conformation I** | **Conformation** **II** | **Conformation** **III** |
| **PDB** | 9IVQ | 9IVR | 9IVS |
| **EMDB** | EMD-60932 | EMD-60933 | EMD-60934 |
| **Data collection** |  |  |  |
| EM equipment | FEI Titan Krios | FEI Titan Krios | FEI Titan Krios |
| Voltage (kV) | 300 | 300 | 300 |
| Detector | Falcon 4i | Falcon 4i | Falcon 4i |
| Data collection mode | counting | counting | counting |
| Pixel size (Å) | 0.725 | 0.725 | 0.725 |
| Energy filter (eV) | 10 | 10 | 10 |
| Electron dose (e^-^/Å^2^) | 40 | 40 | 40 |
| Defocus range (mm) | -1.0 ~ -2.2 | -1.0 ~ -2.2 | -1.0 ~ -2.2 |
| Images | 3,381 | 3,381 | 3,381 |
| **Data processing** |  |  |  |
| Software | cryoSPARC | cryoSPARC | cryoSPARC |
| Number of final used particles | 442,651 | 296,402 | 161,792 |
| Symmetry | D2 | D2 | D2 |
| Map resolution (Å) | 2.66 | 2.80 | 2.97 |
| **Model refinement statistics** |  |  |  |
| Total built residues | 2460 | 2460 | 2460 |
| Model-map-fit CC | 0.87 | 0.80 | 0.82 |
| R.m.s.d. |  |  |  |
| bonds (Å) | 0.004 | 0.004 | 0.007 |
| angles (°) | 0.770 | 0.820 | 1.047 |
| **Molprobity statistics** |  |  |  |
| Molprobity score | 2.45 | 2.40 | 2.67 |
| Ramachandran plot |  |  |  |
| Favored (%) | 94.86 | 95.48 | 94.24 |
| Allowed (%) | 5.14 | 4.52 | 5.72 |
| Disallowed (%) | 0.00 | 0.00 | 0.04 |
| Rotamer outliers (%) | 4.45 | 3.47 | 2.92 |
| Clash score | 12.45 | 14.87 | 27.27 |
| Average B-factor (Å**^2^)** | 125.40 | 156.75 | 159.37 |

**Table S2. The interaction residues between CHIKV-43 and the NTF2L domain.**

| The interaction residues between the N-terminus of CHIKV-43 and the NTF2L domain of G3BP1 | | | |
| --- | --- | --- | --- |
| # | CHIKV-43 (chain L) | Interaction | NTF2L (chain H) |
| 1 | Ile473 | Hydrophobic interaction | *Pro6, Leu10, Val11, Phe124* |
| 2 | Thr474 [N] | Hydrogen bond | *Asn122 [O]* |
| 3 | Thr474 [O] | Hydrogen bond | *Phe124 [N]* |
| 4 | Phe475 | Hydrophobic interaction | *Val11, Phe15, Phe33, Leu114, Phe124* |
| 5 | Gly476 [N] | Hydrogen bond | *Phe124 [O]* |
| 6 | Asp477 [N] | Hydrogen bond | *Arg32 [O]* |
| 7 | Phe478 | Hydrophobic interaction | *Leu22, Met29, Arg32, Phe33* |
| 8 | Phe478 [O] | Hydrogen bond | *Lys123 [NZ]* |
| 9 | Glu482 | Ionic bond | *Arg32* |
| 10 | Glu484 | Ionic bond | *Arg17* |
| The interaction residues between the C-terminus of CHIKV-43 and the NTF2L domain of G3BP1 | | | |
| # | CHIKV-43 (chain L) | Interaction | NTF2L (chain A) |
| 1 | Leu491 | Hydrophobic interaction | *Pro6, Leu10, Val11, Phe124* |
| 2 | Thr492 [N] | Hydrogen bond | *Asn122 [O]* |
| 3 | Thr492 [O] | Hydrogen bond | *Phe124 [N]* |
| 4 | Phe493 | Hydrophobic interaction | *Val11, Phe15, Phe33, Leu114, Phe124* |
| 5 | Gly494 [N] | Hydrogen bond | *Phe124 [O]* |
| 6 | Asp495 [N] | Hydrogen bond | *Arg32 [O]* |
| 7 | Phe496 | Hydrophobic interaction | *Leu22, Met29, Arg32, Phe33* |
| 8 | Phe496 [O] | Hydrogen bond | *Lys123 [NZ]* |
| 9 | Glu500 | Ionic bond | *Arg32* |
| 10 | Asp502 | Ionic bond | *Arg17* |

**Table S3. The interaction residues between the NTF2L domains.**

| The interaction residues between the NTF2L domains in the compact conformation | | | |
| --- | --- | --- | --- |
| # | NTF2L (chain A) | Interaction | NTF2L (chain H) |
| 1 | *Thr75 [O]* | Hydrogen bond | *Asn101 [ND2]* |
| 2 | *Arg78 [NH1]* | Hydrogen bond | *Asn69 [OD1]* |
| 3 | *Arg13* | Ionic bond | *Asp28* |
| The interaction residues between the NTF2L domains in the extended conformation | | | |
| # | NTF2L (chain G) | Interaction | NTF2L (chain F) |
| 1 | *Met1 [O]* | Hydrogen bond | *Arg17 [NH2]* |
| 2 | *Glu4 [OE2]* | Hydrogen bond | *Thr75 [N]* |
| 3 | *Glu4 [OE1]* | Ionic bond | *His74 [ND1]* |
| 4 | *Lys5 [N]* | Hydrogen bond | *Asn24 [OD1]* |
| 5 | *Lys5 [O]* | Hydrogen bond | *Asn72 [ND2]* |
| The interaction residues between NTF2L domains in the upper and lower rings | | | |
| # | NTF2L (chain A) | Interaction | NTF2L (chain N) |
| 1 | *Asn24 [OD1]* | Hydrogen bond | *Arg78 [NH1]* |
| 2 | *Asn69 [O]* | Hydrogen bond | *Arg13 [NH2]* |
| 3 | *Asn72 [OD1]* | Hydrogen bond | *Arg78 [NH2]* |
| 4 | *Asn101 [ND2]* | Hydrogen bond | *Val80 [O]* |
| 5 | *Asn101 [OD1]* | Hydrogen bond | *Val80 [N]* |

**Tables S4. Primers used in this study.**

| **Name** | **Sequence** |
| --- | --- |
| IFN-β-F | TTGCTCTCCTGTTGTGCTTC |
| IFN-β-R | AAGCCTCCCATTCAATTGCC |
| GAPDH-F | GGAGCGAGATCCCTCCAAAAT |
| GAPDH-R | GGCTGTTGTCATACTTCTCATGG |
| NTF2L-F | TTTTCAGGGCGCTAGCATGGTGATGGAGAAGCCTAG |
| NTF2L-R | TGGTGGTGCTCGAGTTAAAAGACCTCATCTTGGTATC |
| CHIKV-43-F | TTCCAGGGGCCCCTGGGATCCGAGACGTTCCCCATCA |
| CHIKV-43-R | AGTCACGATGCGGCCGCTCGAGTTAATGGTGATGGTGATGGTGCGTGGACCAGTCGCT |
| CHIKV-43 mut-R | GTGCGTGGACCAGTCGCTATCTGTCAAATCAGCCACTGCACCGGGTAGGAAGT |
| mCherry-F | AAGGATGACGATGACAAGCTTGTGAGCAAGGGCGAGGAGGA |
| mCherry-R | CAGGGATGCCACCCGGGATCCTTACTTGTACAGCTCGTCCATGC |
| mCherry-nsP3-F1 | GCATGGACGAGCTGTACAAGGCACCATCGTACCGGGTAAA |
| mCherry-nsP3-R1 | TTTACCCGGTACGATGGTGCCTTGTACAGCTCGTCCATGC |
| mCherry-nsP3-R2 | CAGGGATGCCACCCGGGATCCTTACCCACCTGCCCT |
| mCherry-43-F1 | GCATGGACGAGCTGTACAAGGAGACGTTCCCCATCACATT |
| mCherry-43-R1 | AATGTGATGGGGAACGTCTCCTTGTACAGCTCGTCCATGC |
| mCherry-43-R2 | CAGGGATGCCACCCGGGATCCTTACGTGGACCAGTCGCTATCT |
| mCherry-nsP3Δ43-F1 | TCTCCTTCGGAGCACCAAGCTGCTCAGACACGGACGACGA |
| mCherry-nsP3Δ43-R1 | TCGTCGTCCGTGTCTGAGCAGCTTGGTGCTCCGAAGGAGA |
| mCherry-nsP3Δ43-R2 | CAAGGCTGGTGGGCACTGGAGTGGCAACTT |

**References**

1. Schulte T, Liu L, Panas MD, Thaa B, Dickson N, Götte B, Achour A, McInerney GM. 2016. Combined structural, biochemical and cellular evidence demonstrates that both FGDF motifs in alphavirus nsP3 are required for efficient replication. Open Biol 6:160078.
2. Pettersen EF, Goddard TD, Huang CC, Couch GS, Greenblatt DM, Meng EC, Ferrin TE. 2004. UCSF Chimera--a visualization system for exploratory research and analysis. J Comput Chem 25:1605-12.
3. Kril V, Hons M, Amadori C, Zimberger C, Couture L, Bouery Y, Burlaud-Gaillard J, Karpov A, Ptchelkine D, Thienel AL, Kümmerer BM, Desfosses A, Jones R, Roingeard P, Meertens L, Amara A, Reguera J. 2024. Alphavirus nsP3 organizes into tubular scaffolds essential for infection and the cytoplasmic granule architecture. Nat Commun 15:8106.
4. Goddard TD, Huang CC, Meng EC, Pettersen EF, Couch GS, Morris JH, Ferrin TE. 2018. UCSF ChimeraX: Meeting modern challenges in visualization and analysis. Protein Sci 27:14-25.
